# Memory augmented recurrent neural networks for *de-novo* drug design

**DOI:** 10.1371/journal.pone.0269461

**Published:** 2022-06-23

**Authors:** Naveen Suresh, Neelesh Chinnakonda Ashok Kumar, Srikumar Subramanian, Gowri Srinivasa

**Affiliations:** PES Center for Pattern Recognition and Department of Computer Science and Engineering, PES University, Bengaluru, Karnataka, India; Victoria University of Wellington, NEW ZEALAND

## Abstract

A recurrent neural network (RNN) is a machine learning model that learns the relationship between elements of an input series, in addition to inferring a relationship between the data input to the model and target output. Memory augmentation allows the RNN to learn the interrelationships between elements of the input over a protracted length of the input series. Inspired by the success of stack augmented RNN (StackRNN) to generate strings for various applications, we present two memory augmented RNN-based architectures: the Neural Turing Machine (NTM) and the Differentiable Neural Computer (DNC) for the *de-novo* generation of small molecules. We trained a character-level convolutional neural network (CNN) to predict the properties of a generated string and compute a reward or loss in a deep reinforcement learning setup to bias the Generator to produce molecules with the desired property. Further, we compare the performance of these architectures to gain insight to their relative merits in terms of the validity and novelty of the generated molecules and the degree of property bias towards the computational generation of *de-novo* drugs. We also compare the performance of these architectures with simpler recurrent neural networks (Vanilla RNN, LSTM, and GRU) without an external memory component to explore the impact of augmented memory in the task of *de-novo* generation of small molecules.

## Introduction

Conventional drug discovery is a resource intensive process, with the average time from clinical testing to market approval taking an estimated 96.8 months and incurring a monetary cost of approximately $2,870 million [1]. Hence, the use of *‘in silico’* (or computational) methods has gathered momentum in aiding the design of drugs [2, 3]. With a rapid increase in the capability of computational resources and availability of copious amounts of data, in addition to the savings of cost and time, artificial intelligence, in particular, has started to play a key role in areas such as drug repurposing [4], computer-aided drug design [5–8] and testing for synthetic accessibility [9]. The COVID-19 pandemic has further underscored the relevance of computational methods to aid in drug discovery and design [10–13]. Among the approaches for *in silico* drug discovery, deep learning models have been successfully adopted to suggest chemically feasible molecules with desired chemical and pharmacological properties [14–17].

A crucial design consideration when using deep learning models for *de-novo* drug discovery is the presence and efficacy of a generative model to produce representations of the drug molecules. A candidate representation could be the 1-D enumeration of characters, in the Simplified Molecular-Input Line-Entry System (SMILES) format. Inspired by the success of Popova et al. (2017), the overall process of drug design has been divided into (i) the generation of candidate SMILES strings and (ii) biasing the generator to generate SMILES strings containing desired properties [18]. This framework uses deep reinforcement learning to bias the generation of novel molecules. The Generator is a neural network that produces molecules in the form of SMILES strings; it is first trained for the task of generation using a set of valid SMILES strings. This model can be an implementation of a generative model that has been used for other string generation tasks such as recurrent neural networks, Variational Autoencoders [19], or Generative Adversarial Networks (GANs) [20, 21]. The strings generated are passed to the Predictor. The Predictor predicts certain properties using the molecular structure. These predicted properties are used to formulate a reward, which is input back to the Generator and is used to bias the generation of molecules resulting in the generated molecules possessing a specific property.

Generated molecules are valid if and only if their corresponding string forms obey the syntax of the SMILES representation. Points of branching in the molecule are denoted by brackets, and this implies that multiple types of brackets must be matched correctly and closed in the molecule generated. A Vanilla RNN, with but a hidden state to use as context, while capable of the task of generation, however, is not adept at capturing this sequence of brackets [22]. This necessitates augmenting the network with a memory element. A recurrent neural network augmented with greater memory potential, is capable of generating valid SMILES strings rife with brackets and rings as the extra memory is used to keep track of the information required to close opening braces over a protracted length of the input, among others, while the hidden state keeps track of the remaining context. We have experimented with three different memory augmented RNNs: the Stack Augmented RNN, the Neural Turing Machine and the Differential Neural Computer [23]. Each of these generators makes use of a different mechanism for the augmented memory. An evaluation of the generated molecules lends insight to the utility of these models in aiding *de-novo* drug generation.

### Contributions of this work

(1) Three RNN-based architectures augmented with external memory for *de-novo* generation of small molecules -
  (a) refactoring of a stack augmented RNN, a version of which has been established to be useful for *de-novo* generation of small molecules [18] and
  (b) adaptations of two recurrent neural network architectures, the NTM and DNC, with external memory to support random access, an advantage over the first-in-last-out access imposed by a stack and establishing their efficacy for the task of generating small molecules.
(2) Three baseline generation models—The Vanilla RNN, GRU and LSTM. We compare the memory augmented architectures to the baseline models to better determine their efficacy.
(3) A comparison of all the generative models in terms of the percentage yield (to quantify the validity of generated molecules) and percentage of overlap with an existing chemical database (to quantify the novelty of generated molecules) to gain insight to the value add offered by each model for *de-novo* drug generation.
(4) A deep-learning-based, character-level CNN Predictor to learn to predict a property associated with the generated SMILES string and bias the Generator to generating strings with the desired property within a reinforcement learning framework.
(5) A comparison of the distributions of the molecules generated by the six models to assess the extent of biasing towards a desired property.
(6) Access to the source code for all components, training data used to train the models presented in this study and the SMILES strings generated by the six models for various experimental conditions, in the spirit of reproducible research [24].

## Materials and methods

**Fig 1:**
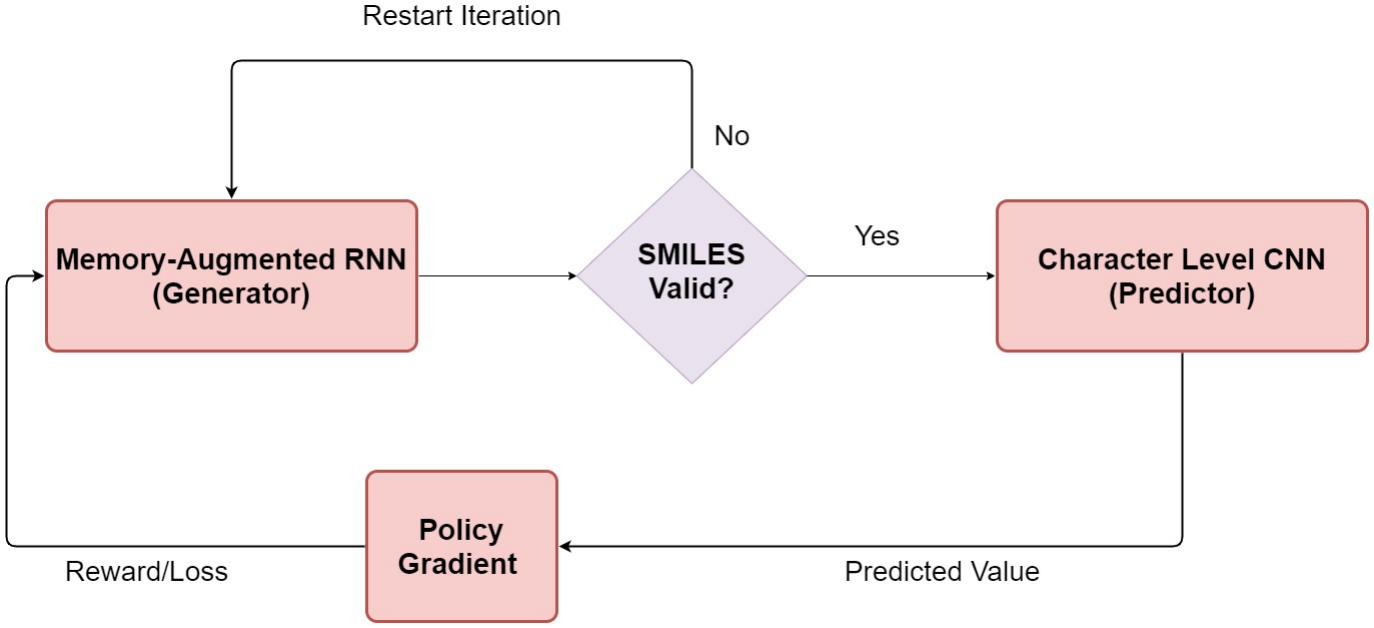
A schematic diagram of the framework.

A schematic block diagram of the framework used in this work is shown in Fig 1. Broadly, there are two major components: (i) a Generator and (ii) a Predictor, whose output is used to bias the memory augmented RNN-based Generator towards a desired property through reinforcement learning. In the generation phase, the molecule generated is implicitly checked against rules of validity, such as valency. If the SMILES representation is valid, the string is input to the Predictor—a character level convolutional neural network (CNN)—for computation of the property of interest. A policy gradient algorithm is used to compute a reward or loss to the Generator for subsequent generation.

These components and the process of biasing the generator are discussed in detail subsequently.

### Data

To demonstrate the efficacy of the models presented, the Generators are required to output valid molecules represented using SMILES strings. For this purpose, we first trained each Generator with SMILES strings for molecules known to exist and independently trained the Predictor to learn a property we desire to predict from the SMILES strings.

The data used to train the Generator and Predictor have been obtained from ChEMBL [25]. ChEMBL lists around 1.6 million compounds. We have sampled a subset of 788, 452 SMILES strings from this database (such that the molecular weight does not exceed 900) for use in training the memory augmented RNN-based models to generate valid molecules. The Generators were validated through the generation of over 4000 SMILES strings. The details of the data used to train the generator are presented in Table 1, and further details are discussed in Section 1 of the S5 File.

**Table 1:** Properties of molecules in the dataset.

Property	Q1	Q2	Q3	Mean	Range
Mol Wt	278.173	320.164	354.138	314.136	798.707
LogP	1.982	2.941	3.858	2.877	20.864
Benzene	1.0	1.0	2.0	1.180	7

We biased the Generators based on two properties: logP and the number of Benzene rings. P represents the partition coefficient and gives an indication of the extent to which a molecule would be absorbed by living tissue or carried away by water [26]. The property logP is computed as the log to the base 10 of the partition coefficient, P. This and the number of Benzene rings can be calculated from the structure of the molecule using RDKit [27]. For the predictor, we sampled 200, 000 SMILES strings from the entire dataset and computed logP values (and the number of Benzene rings) for these strings, which were used as the target (or ground truth). An 80–20 split was the train/test split used to validate the predictor. The properties mentioned above are used as placeholders for any property one may wish to compute from the generated molecule in the actual process of designing *de-novo* drugs. The Generator is biased to minimize the property and maximize the property in two separate experiments, conditioned on the SMILES strings generated being valid, as a proof of concept of the adaptability of the Generator. The validity of the SMILES strings generated, with and without the property bias, has been tested using RDKit.

### Generators

At the core of the three Generators in this study is a memory augmented RNN. RNNs have successfully generated strings due to their ability to learn inter-dependencies and transform data over protracted lengths of the input. A limitation is that it is unable to perform well at tasks that involve repetition or keeping track of specific attributes of the data.

#### Stack augmented RNN

**Fig 2:**
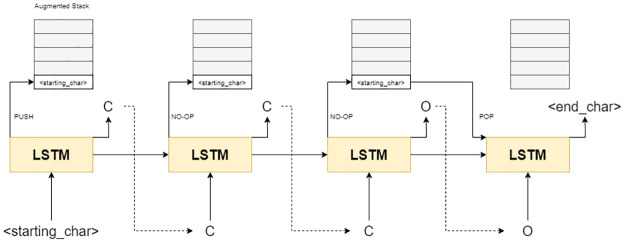
A schematic diagram of the generation process with StackRNN.

Joulin et al. proposed the augmentation of a push-down stack to aid as external memory to the RNN, thus enhancing its capability to keep track of tokens in a long string [22]. This is demonstrated in Fig 2.

Its ability was accentuated when presented with the Dyck language, which consists of strings where all the brackets are matched [28]. This rationale motivated the use of memory augmented RNNs such as the StackRNN in the field of drug design for the generation of SMILES strings with promising success [18].

The advantage of a StackRNN over an LSTM in the task of drug generation as demonstrated drug generation architecture proposed by by [18] inspired us to pursue memory augmented architectures that were more capable than the stackRNN. To work towards this, we consider the architectures proposed by Graves et al., the Neural Turing Machine (NTM) [29, 30], and its extension, the Differentiable Neural Computer (DNC) [23, 31], for *de-novo* generation of molecules. The NTM and DNC architectures consist of an external memory that can be accessed randomly by using an address—a vector of weights—corresponding to each memory location. Memory accesses are decided by a controller—a deep neural network—which, in our case, is a Long Short Term Memory (LSTM) recurrent neural network.

#### Neural turning machine

**Fig 3:**
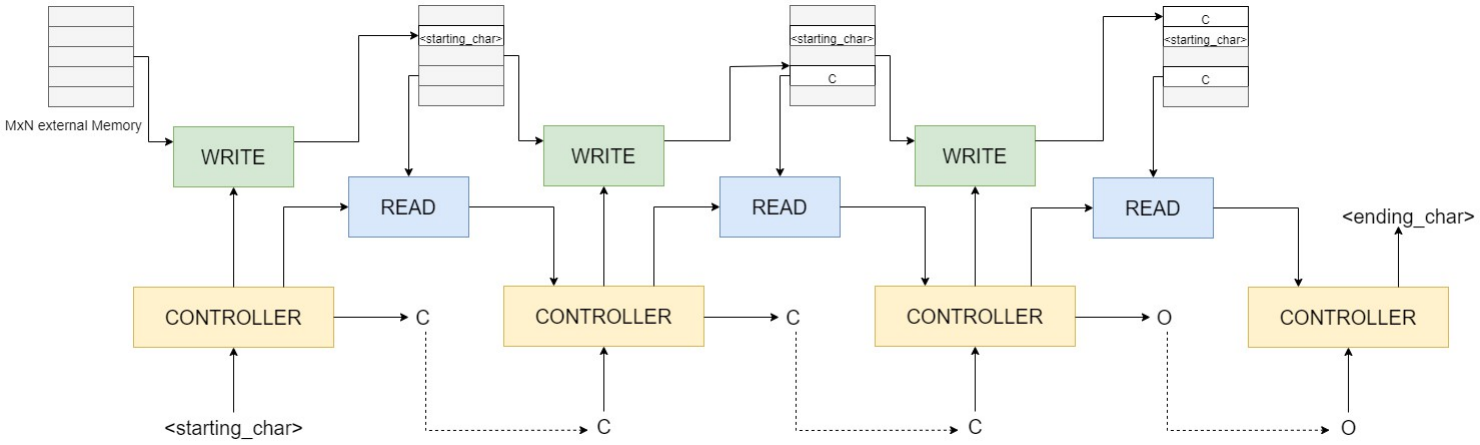
A schematic diagram of the generation process with NTM.

The NTM comprises a controller and an external memory, a matrix of *N* locations of vectors of size *M*, as shown in Fig 3.

The external memory in NTM is accessed by read heads that read from memory and write heads that write content into memory. The LSTM controller is trained with the external memory in an end-to-end manner. This type of memory helps in the task of sequence generation because a portion of the content is not lost (*vis-a-vis* a stack where it is lost when the top element of the stack is popped). This, in turn, aids the use of more information which can be used repeatedly in the generation of tokens (for *de-novo* generation of molecules, these can be functional groups, etc.).

#### Differentiable neural computer

**Fig 4:**
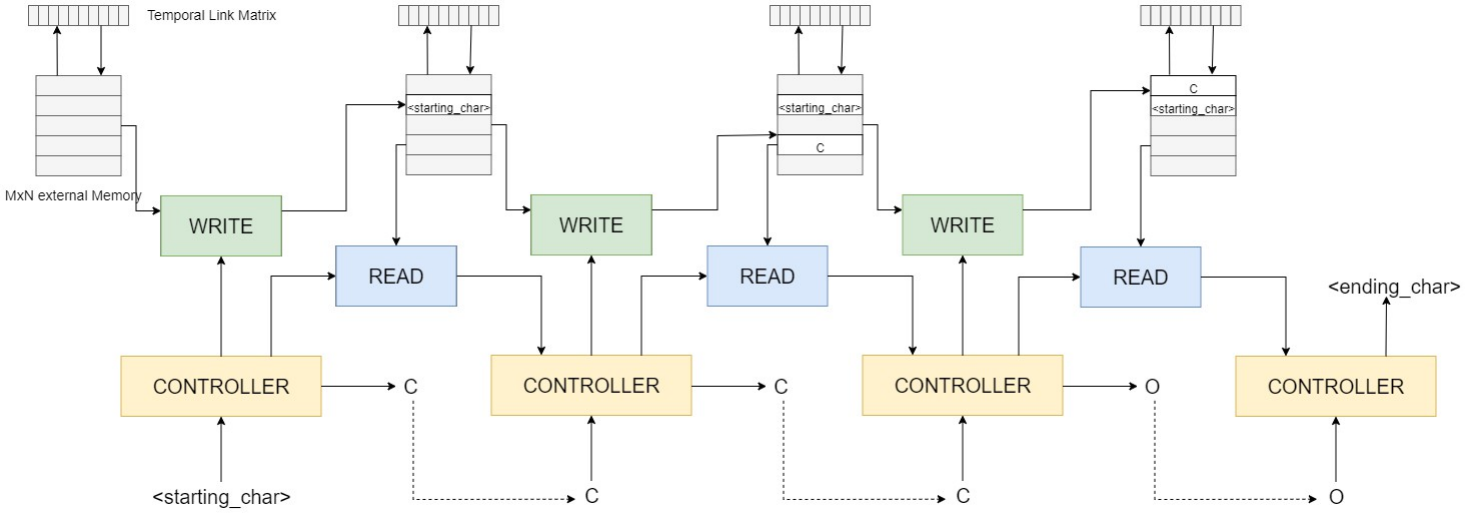
A schematic diagram of the generation process with DNC.

The DNC gets its name because the function that produces the weights for the read and write heads is differentiable. This allows the use of gradient descent, etc., procedures to find the most appropriate weights for each function: read and write. The DNC, an extension of the NTM, has an interface vector between the controller and read-write heads (see Fig 4).

This interface has three components: the content lookup, collected in a key vector and corresponds to the content of each vector, a temporal link matrix that keeps track of the order of writes, and a memory allocation that records the extent of usage, preventing overwrites. These sub-components can potentially enhance the quality of sequences generated.

#### Baseline generators

We compare the memory augmented RNNs with three baseline models. They are the Vanilla/simple RNN [32], Gated Recurrent Unit network (GRU) [33], and the Long Short Term Memory network (LSTM) [34]. Vanilla RNNs have issues with remembering information over a protracted length of time. LSTM’s were designed to overcome these problems and in addition to this, have been demonstrated to learn context sensitive languages [35]. Context sensitive languages require counting, and the linear units of the LSTM can be used for this purpose. The GRU is a more recent network, which is similar to the LSTM.

Both the NTM and the DNC Generators can be expected to perform at least as well as the StackRNN. This is because the memory with random access in NTM and DNC can be used as a stack [36]. We also hypothesize the StackRNN to perform at least as well as the GRU/LSTM baseline models, as the StackRNN can be considered to be a GRU/LSTM with extra memory. The Vanilla RNN is usually outperformed by the GRU and LSTM, and we expect the same to hold here.

#### Training and testing the generator

**Fig 5:**
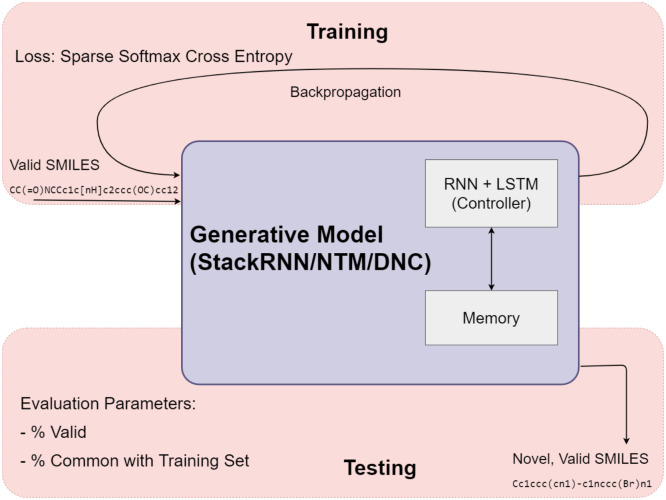
Training and testing workflows for the generator.

A schematic diagram of the workflow for training and testing the Generator is presented in Fig 5. In the training phase, valid SMILES strings are input to the Generator (Baseline models, StackRNN, NTM or DNC). The Generator is expected to learn the patterns in the valid SMILES strings to predict the next most likely character (that could include parenthesis, etc.). This is checked against the ground truth (actual string) to compute a loss using Sparse Softmax Cross Entropy. The loss computed is used to adjust the weights of the RNN through a process of backpropagation.

In the testing phase, the Generator is expected to output SMILES strings that are valid and novel. For a given iteration number, the Generator’s performance is evaluated based on the percentage of valid strings generated of a pool of molecules output by the Generator. RDKit is used to compute the validity. To assess the Generator’s ability to generate novel molecules, the percentage of overlap of the molecules output by the Generator with those used during the training phase is computed. The higher the percentage of valid and novel molecules, the better the ability of the Generative model.

### Predictor

The Predictor takes as input a valid molecule generated by one of the generators. It must predict the property of interest. The RL framework uses the value of the property predicted to generate a reward or loss to drive the Generator to output molecules with the desired property. Deep neural networks have been demonstrated to be capable of performing this task [37]. In our system, the Predictor is a character-level Convolutional Neural Network (CNN).

#### Training and testing the predictor

**Fig 6:**
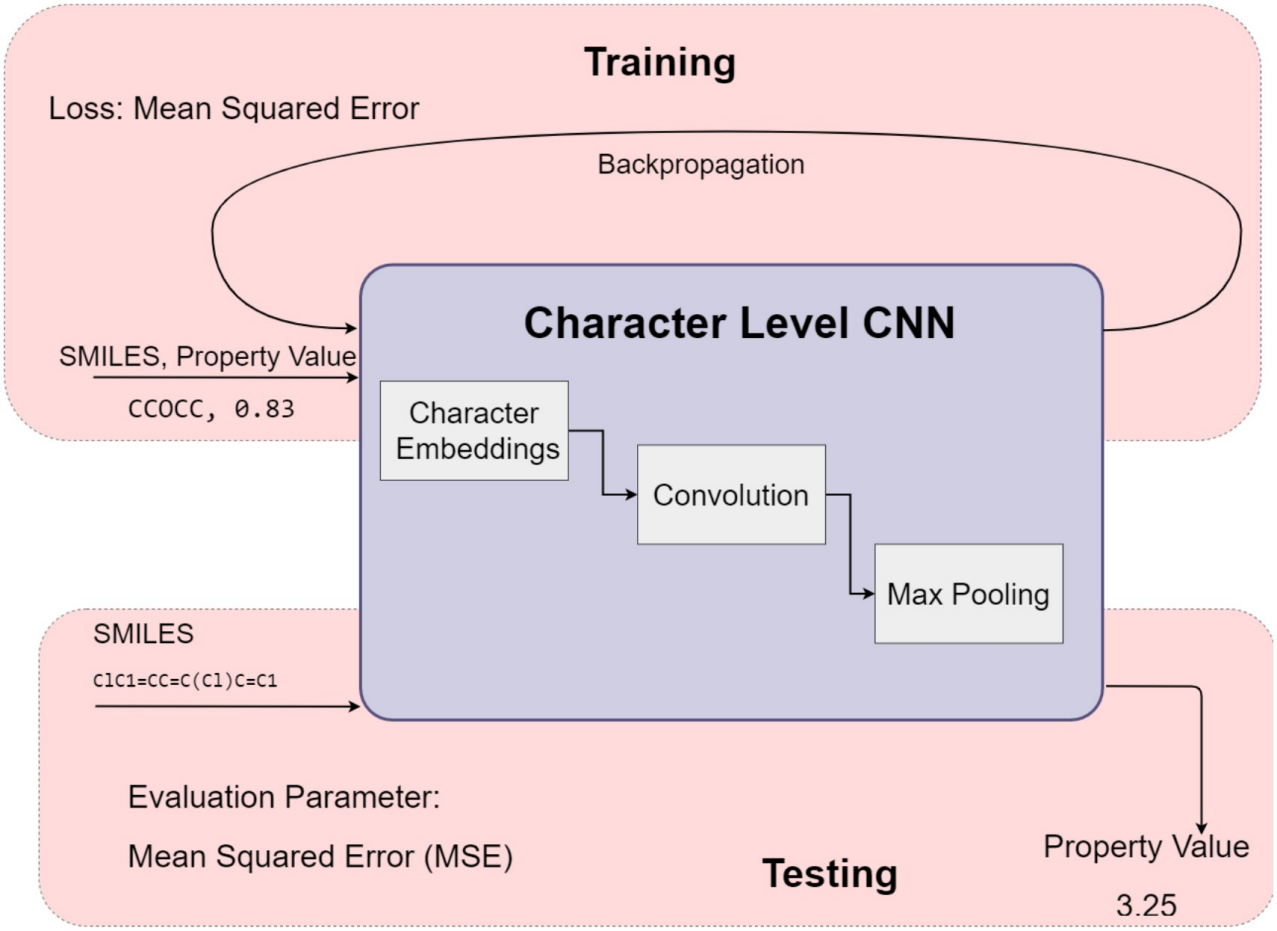
Training and testing workflows for the predictor.

A schematic diagram of the workflow for training and testing the Predictor is presented in Fig 6. Since the Predictor is designed to bias a Generator to generate SMILES string with the desired property, in the training phase, the input comprises 160, 000 valid SMILES strings with the value of the property corresponding to each input molecule.

The input size to the character-level CNN is 121 as 98% of the strings lie within that range. (In fact, the average string length in the training set is about 36) This is followed by character embeddings, with the embedding length set to 46. (This corresponds to the number of tokens (45) permitted in SMILES + 1 should a character outside of the tokens used in the experiment be present in the string input to the predictor).

The CNN comprises 5 layers; convolution and max-pooling are applied to each of the layers. Convolution is the mapping of an input matrix to an output matrix of equal or lower dimension by applying some function over a particular size of the sub-matrix. Max Pooling is the selection of a maximum value over a sub-matrix [38]. The network is flattened and given dense layers with ReLu activation and a dropout probability of 0.5. The Adam optimizer [39] is used to learn the value of the property for a given SMILES string. The loss function is the mean squared error, computed as the mean of the square of the difference between the predicted value, *p*, and target value for the property of interest. The weights of the CNN are adjusted through a process of backpropagation to minimize the loss.

Since the training and validation of the Predictor are run independently, in the testing phase, 40, 000 valid SMILES strings that have not been used in the training phase are input to the Predictor. The property value computed by the Predictor is evaluated against the property value computed using RDKit for those input SMILES strings. This is followed by the computation of the mean squared error (MSE). Lower the MSE on the validation data, the better.

### Reinforcement Learning (RL) to bias the generator

To bias the Generator to output valid SMILES strings with the desired property, a reward is constructed using the predicted value for the property (see Algorithm 1). For every string generated, the value, *p*, of the property of interest is computed. A scaling factor *s* is applied to generate a loss. The total loss is computed as the sum of the predicted loss and cross-entropy loss. This is run through multiple batches of SMILES strings generated by the current model to obtain optimal weights. The reward thus computed is used by the Generator to bias the generation of molecules.

Algorithm 1 delineates the steps for the Generator to output SMILES strings to be used in RL. For any given state of the output from the Generator, a random number is generated. If the random number exceeds *ϵ*, a preset threshold that determines stochasticity, a character is drawn from the current model output. If not, the distribution is sampled *k* times, and a list of unique characters is obtained. Then, a random choice is made between the characters in this list. Either of these preceding steps determines the next character. This stochasticity ensures that we encourage exploration by increasing the odds of choosing the lower probability characters.

The reward assigned from the predicted output, *p*, plays a crucial role in the process. For the purpose of maximisation (or minimization) of a property, the reward used is an exponential function of the predicted output value as delineated in Algorithm 2. Rewards are finally maximized using a policy gradient approach.

**Algorithm 1**: String Generation

 **Input**: drawFromDist(*n*, *k*): draw *n* characters from distribution *k*,

 *ϵ*: Threshold for sampling 1 or n characters from the currentModelOutput,

 batch_size: number of strings to be generated per batch,

 currentModelOutput: the softmax output of current model,

 getSoftmax(char): softmax distribution from the generative model for next_char,

 uniq(input_list): returns a list of uniq values in input_list

 **Output**: gen_SMILES: Generated set of SMILES strings by current model

1 **for** *i* ← 0 to *batch*_*size* **do**

2  next_char = starting_char

3  **while** next_char ≠ *end_char* **do**

4   *gen*_*SMILES*[*i*].*append*(*next*_*char*)

5   *nextCharacterDistributionFromModel* ← *getSoftmax*(*next*_*char*)

6   *next*_*char* ← *GetNextCharacter*(*nextCharacterDistFromModel*)

7 **Function** GetNextCharacter(*currentModelOutput*):

8  **if** *drawFromDist(1, uniform(0,1))* > *ϵ* **then**

9   next_char ← drawFromDist(1, currentModelOutput)

10  **else**

11   next_char_candidates ← drawFromDist(n, currentModelOutput)

12   unique_next_char_candidates ← uniq(next_char_candidates)

13   next_char ← randomChoice(unique_next_char_candidates)

14  **return** next_char

**Algorithm 2**: RL Model Weights Optimization

 **Input**: p: predicted value from the predictive model, s: scaling factor to generate loss, optimizer: gradient descent algorithm to train network

 **Data**: gen_SMILES: Generated set of SMILES strings by current model *x*

/*cross_entropy_losscalculatedascrossentropybetweenone-hotselectedcharacterandsoftmaxoutput*/

1 **for** *all batches of gen_SMILES* **do**

2  *predicted*_*loss* ← *e*^(p*s)^      //Lossattributedtovaluepredicted

3  *total*_*loss* ← *predicted*_*loss* + *cross*_*entropy*_*loss*

4  *optimizer*(*model*, *total*_*loss*)

In this manner, the RL framework uses the predicted value to train the Generator to produce molecules biased towards a particular property. This is based on the REINFORCE algorithm which uses the expected value of reward [40].

## Results

Evaluation of the performance of the Generator, Predictor and the outcome of RL-based biasing of the Generator to favor a property are presented and discussed subsequently.

### Generator

The Generators were evaluated on two fronts: (i) the efficacy of the Generator to output valid SMILES strings and (ii) the percentage of the valid SMILES strings that are novel.

A summary of the parameters used for the Generators is presented in Table 2. For the baseline models, the same parameters as the StackRNN were used, apart from the external memory.

**Table 2:** A summary of the parameters used for the memory augmented generators.

Parameter	StackRNN	NTM	DNC	Rationale
Embedding length	1024	1024	1024	Explained in Supplementary Material (SM) Table 2 (for NTM); we kept the length consistent across all Generators.
Number of computational (LSTM) units	1024	1024	1024	Explained in Supplementary Material (SM) Table 2 (for NTM); we kept the number of units consistent across all Generators.
Size of the external memory (in units of embedding length)	200	200	200	Maximum length of the SMILES string in training was 120. To avoid overflow and to ensure a fair comparison across models, the depth of the stack for StackRNN and size of the external memory for NTM and DNC, were kept the same and set to 200
Batch size	26	38	26	Based on the computational feasibility for each Generator
Total batches for testing	200	200	200	We wanted to generate over 4000 strings for each Generator. Hence, fixed the number of batches at 200
Other parameters	SM 1.1	SM 1.2	SM 1.3	Model-specific and minor parameters have been detailed in the SM for brevity

We generated SMILES strings for over 4, 000 small molecules using each Generator model and computed the percentage of valid molecules among them. The validity of SMILES strings is determined by converting the string into its structural representation [41]. An unsuccessful attempt to convert a string to its structural representation indicates that the string does not correspond to a valid molecule. Table 3 contains the percentage of valid SMILES strings that are generated by each model at every 5000 iterations.

**Table 3:** Percentage of valid strings generated by each memory augmented model at a particular iteration.

Number of Iterations	Percentage of valid strings		
	StackRNN	NTM	DNC
5000	84.8	**87.2**	81.7
10000	88.7	**89.0**	86.2
15000	91.7	**92.2**	90.9
20000	92.6	**93.5**	91.8
25000	92.7	**93.3**	92.3
30000	93.5	**93.9**	91.8
35000	93.9	94.2	**94.5**
40000	94.1	**94.9**	94.1
45000	93.1	94.7	**94.8**

We note that the percentage of valid strings, unbiased towards any property, are similar across Generators, with the NTM and DNC presenting with a marginally higher percentage of valid strings as the iterations increase. Overall, we note a 94% validity, implying a reasonable yield of small molecules.

Next, the novelty of the valid molecules is determined. This is done by computing the percentage of SMILES strings that are common between those generated by the Generators and the strings in the training data (i.e., 788, 452 small molecules from ChEMBL). (The Common % is not very different even when compared with the entire data of over 1.6 million strings: these numbers are reported in Section 3.8 of the S5 File). We present these together with the computation of the first three quartiles with respect to the logP property, for which the models have not (yet) been biased. A comparison of the logP values and percentage of valid SMILES strings and percentage of SMILES strings that are common with the training data for each of the Generators is presented in Table 4.

**Table 4:** A comparison of the generators, unbiased for any property.

Model	Q1	Q2	Q3	Mean	Valid %	Common %	Average Length	Median SA Score
StackRNN	1.91	2.96	3.96	2.9	93.1	4.85	36.01	2.56
NTM	1.84	2.86	3.8	2.78	94.71	8.2	36.06	2.52
DNC	1.71	2.74	3.73	2.69	94.77	7.06	35.68	2.53
GRU	1.93	2.95	3.97	2.95	82.43	2.12	36.26	2.76
LSTM	1.89	2.89	3.9	2.88	92.58	5.15	36.28	2.7
Vanilla RNN	1.5	2.64	3.78	2.67	31.23	3.6	36.32	2.83

We note that overall, about 92% of the SMILES strings output by the Generator have no overlap with the training data. It is interesting that the percentage of common SMILES strings is least for StackRNN (around 5%).

The average length of the SMILES strings generated by the three models is about 36 and comparable to the average length of SMILES strings in the training data (which is also about 36).

The synthetic accessibility (SA) score gives an estimate of the ease of synthesizing a compound [42]. The SA score lies in (1,10) and a score closer to 1 implies the compound is easier to synthesize. The median SA score for the unbiased Generators is about 2.5 across the three models.

We also see in comparison to simpler RNN architectures without external memory, the memory augmented models were able to generate valid SMILES strings more consistently, especially with respect to the performance of the Vanilla RNN (31.23%) and the GRU (82.43%). The LSTM (92.58%) performs better than the GRU, and is comparable to the StackRNN.

#### Lower computational units experiments

We can see that with 1024 units, the LSTM performs comparably to the StackRNN. This seems to indicate that the extra memory of the StackRNN did not have any effect. One possible reason for this, is that for our problem and dataset, the amount of memory required is satisfied by the 1024 computational units, and thus, the extra memory of the StackRNN was futile. To validate this hypothesis, we have run experiments with lower computational units and memory. We compare only the LSTM with the StackRNN as the StackRNN uses LSTM units, and it performed the worst out of the augmented models. Hence, the StackRNN can be considered the smallest improvement over the baseline LSTM (as opposed to the NTM and DNC which have some extra capabilities). We present the percentage of valid strings with 16, 32, 64 and 128 computational (LSTM) units in Tables 5–8 respectively. Additionally, for a constant number of computational units, we vary the stack width of the StackRNN, to vary the total amount of memory available. The rest of the parameters are the same. Tables 5–8 present the percentage of valid strings for various computational (LSTM) units with the stack width represented in parenthesis.

**Table 5:** Percentage of valid strings generated by a model with 16 computational (LSTM) units at a particular iteration.

Number of Iterations	Percentage of valid strings			
	LSTM	StackRNN(4)	StackRNN(8)	StackRNN(16)
5000	4.4	**5.0**	3.9	4.4
10000	9.8	9.4	**11.3**	10.5
15000	13.8	13.2	14.2	**19.2**
20000	13.8	16.7	**20.6**	18.2
25000	20.8	18.8	**21.5**	17.7
30000	17.8	19.2	**24.8**	22.8
35000	23.2	24.2	26.8	**28.2**
40000	20.5	23.9	**32.8**	27.7
45000	23.8	23.1	25.2	**31.7**

**Table 6:** Percentage of valid strings generated by a model with 32 computational (LSTM) units at a particular iteration.

Number of Iterations	Percentage of valid strings		
	LSTM	StackRNN(32)	StackRNN(512)
5000	18.1	**22.4**	13.4
10000	27.5	29.8	**33.9**
15000	34.9	**36.4**	36.1
20000	41.7	**45.8**	40.8
25000	38.5	46.8	**47.9**
30000	39.5	53.1	**56.5**
35000	44.0	**51.6**	50.2
40000	47.7	49.7	**56.1**
45000	46.7	54.8	**58.5**

**Table 7:** Percentage of valid strings generated by a model with 64 computational (LSTM) units at a particular iteration.

Number of Iterations	Percentage of valid strings	
	LSTM	StackRNN(64)
5000	38.8	**44.0**
10000	51.4	**52.6**
15000	58.7	**64.5**
20000	65.1	**69.8**
25000	66.0	**70.6**
30000	**66.0**	65.7
35000	67.8	**73.0**
40000	**69.9**	68.2
45000	70.8	**75.2**

**Table 8:** Percentage of valid strings generated by a model with 128 computational (LSTM) units at a particular iteration.

Number of Iterations	Percentage of valid strings			
	LSTM	StackRNN(32)	StackRNN(128)	StackRNN(512)
5000	59.0	56.4	62.8	**68.4**
10000	72.2	**72.6**	70.1	68.1
15000	73.0	75.4	**78.2**	75.3
20000	76.9	72.8	**79.4**	77.7
25000	78.5	78.5	77.6	**79.5**
30000	77.5	77.1	79.8	**83.1**
35000	79.2	80.5	80.9	**81.7**
40000	81.7	82.8	83.0	**84.0**
45000	**85.8**	85.3	81.7	79.9

From these experiments and our earlier results, we can note the following. From 16 units to 64 units, augmented memory does help in increasing validity percentage. However, for 128 units, the increase in stack memory seems to have no effect, with all the models performing similarly. Along with our results for the models with higher computational units where the LSTM performed comparably to the StackRNN, we can conclude for our problem and dataset, that beyond a certain number of LSTM units (128 in our case), the benefit of augmented memory is negligible.

### Predictor

The performance of the Predictor was quantified using the mean square error (MSE), where the error is the difference between the value predicted for a property of interest for an input SMILES string and the ground truth value of the property for that string. The properties used as a proof of concept are logP and the number of Benzene rings.

For each property, the Predictor was trained on a random sample of 200, 000 (of the original 788, 452) valid SMILES strings with a 80 − 20 train-test split. This model achieved an MSE of 0.079 for logP. (The results of the Predictor for predicting the number of Benzene rings is presented in Section 3.7 of the S5 File. These properties are used as a proof-of-concept of the effectiveness of the framework to bias the Generators for any other property, whether continuous or discrete, that is of interest in an actual application. Without any loss of generality, the discussion in this paper will be restricted to the biasing of Generators for logP).

The same Predictor, i.e., character-level CNN, was used in conjunction with all the Generators for the purpose of reinforcement learning.

### Reinforcement learning to bias the generator

How effective is the RL framework in biasing the Generators? To what extent do the Generators output strings that are biased towards a property? How does the biasing affect the performance of the Generators in terms of the percentages of valid strings and novel strings generated? To answer these pertinent questions and assess the quality and distribution of molecules generated by the models, various metrics were calculated. Among these, the percentage of valid molecules and percentage of common molecules when compared to the training data-set discussed earlier are two important measures. Besides this, the median synthetic accessibility score are reported [43]. Further, we report the average length of the strings generated by a biased model to ensure the Generators continue to generate strings that are not only valid and novel, but comparable in length to those in the training data.

**Fig 7:**
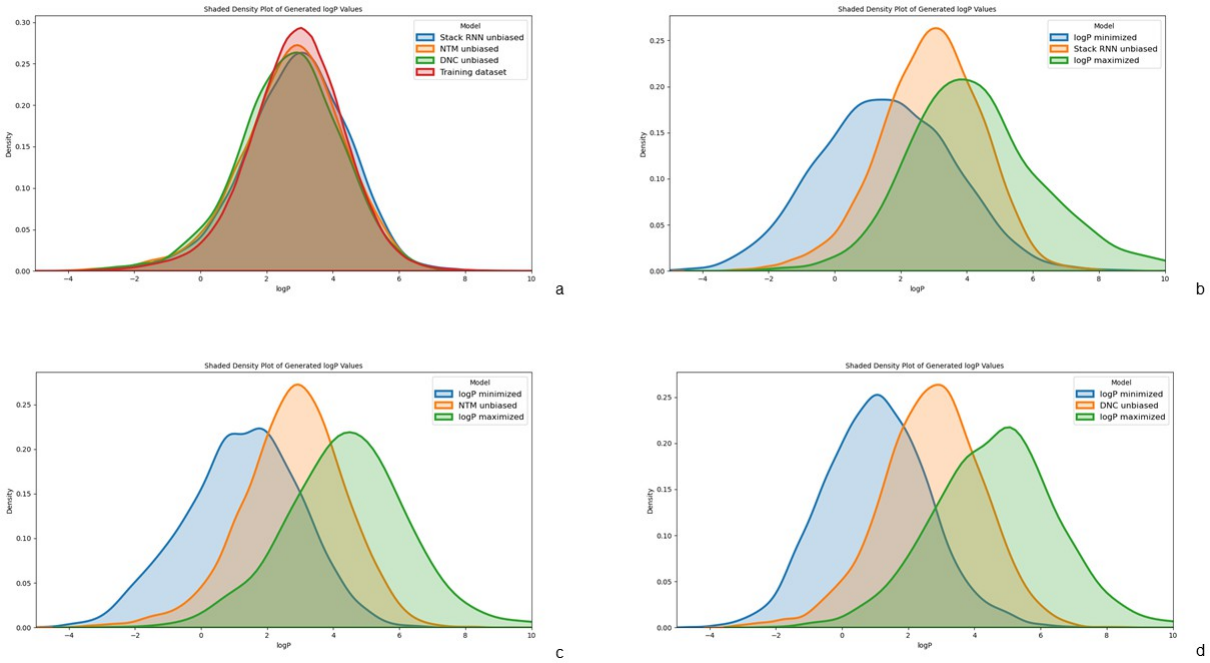
A comparison of the Unbiased and Biased Memory Augmented Generators through a density plot of log P values for (a) the training data and each of the three generators: StackRNN, NTM and DNC without any biasing, (b) Unbiased StackRNN and StackRNN with biasing for both minimizing log P values and maximizing log P values, (c) Unbiased NTM and NTM with biasing for both minimizing log P values and maximizing log P values and (d) Unbiased DNC and DNC with biasing for both minimizing log P values and maximizing log P values.

The number of SMILES strings generated by each of the models is sufficiently large, greater than 1000. As can be seen in Fig 7, density plots of logP computed for strings generated by each of the Generators resemble normal distributions that are unimodal.

For the unbiased Generator, the distribution of both the training data molecules and the generated molecules resemble normal distribution indicating that the model has generalised well (see Fig 7). The trained models all have medians around or lower than that of the training dataset (2.95). This may imply that the strings the model has learned to generate fall within that region. The distribution after biasing for each of the models shows a lower mean logP for the minimized models and a higher mean logP for the maximized models, confirming there is a biasing in the desired direction.

#### Minimizing logP

From the graphs in Fig 7, the biasing is evident in all three memory augmented Generator models. The quartiles for logP are reported in Table 9. Comparing these values with those of the corresponding unbiased models in Table 4, we note the statistics are consistent with the graphs. Each of the quartiles and the means of the logP values of molecues output by the three generators present with lower logP values when biased to minimize logP. Even among them, the DNC is seen to generate molecules with relatively lower logP values compared to the StackRNN and NTM.

**Table 9:** A comparison of the generators biased for logP.

Bias	Model	Q1	Q2	Q3	Mean	Valid %	Common %	Average Length	Mean SA Score
Min logP	StackRNN	0.13	1.52	2.96	1.55	96.56	1.19	38.83	3.08
	NTM	0.11	1.31	2.45	1.24	96.15	4.02	29.14	2.82
	DNC	**−0.05**	**1.01**	**2.06**	**1.03**	92.69	2.68	35.04	2.95
	GRU	0.33	1.22	2.08	1.2	75.77	3.92	31.05	2.96
	LSTM	1.25	2.04	2.99	2.11	86.05	0.35	35.90	3.16
	Vanilla RNN	1.84	2.01	2.33	2.09	98.5*	0*	11.97	2.32
Max logP	StackRNN	2.91	4.11	5.48	4.32	90.49	1.53	41.59	2.87
	NTM	3.25	4.46	5.65	4.66	95.5	1.48	36.05	2.84
	DNC	**3.41**	**4.70**	**5.86**	**4.69**	94.62	0.68	37.94	2.97
	GRU	2.99	4.16	5.55	4.27	88.47	2.85	35.08	3.2
	LSTM	3.06	4.47	5.82	4.5	86.79	1.61	35.18	2.97
	Vanilla RNN	2.73	2.73	2.73	2.73	99.55	0.075	11.02	2.43

* Out of 4000 strings generated, only 153 were unique.

**Fig 8:**
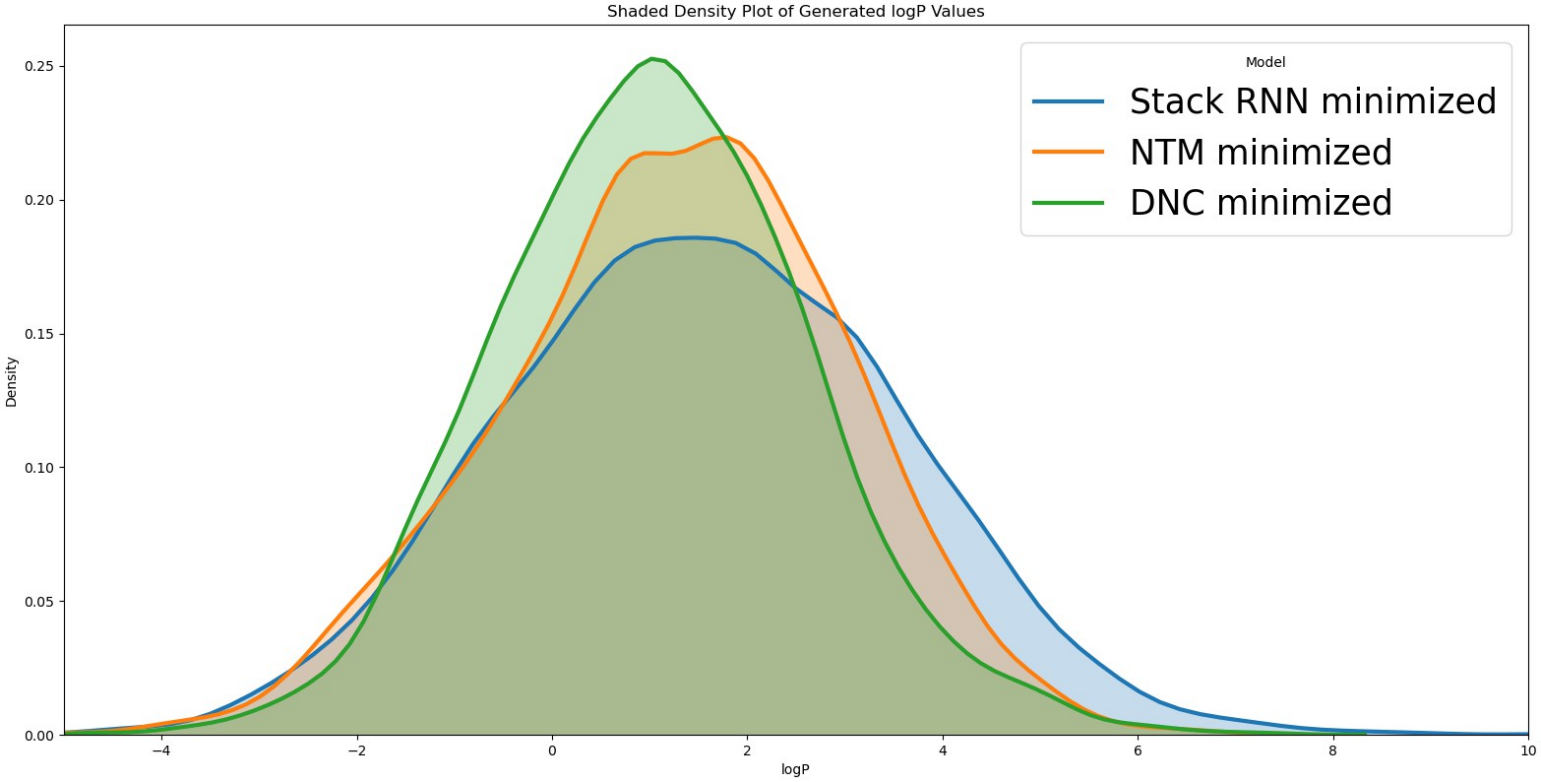
A comparison of the external memory augmented generators biased to minimize log P.

From Fig 8, we note the StackRNN has a denser right tail and thus produces a more significant portion of molecules with a higher value of logP compared to the NTM and the DNC.

Among the baseline models, Vanilla RNN and LSTM failed to minimize logP to the same extent as the memory augmented RNNs, with both models having largely unaffected distributions by the biasing process. The performance of GRU, on the other hand, was comparable to that of memory augmented RNN’s, with the average logP of the generated strings being less than that of the strings generated by StackRNN.

The percentage of strings generated that are valid from the models that minimize logP are fairly different from their corresponding unbiased models. StackRNN and NTM have a high yield with 96% of the strings generated being valid, whereas DNC has a yield of over 92% valid strings.

Among the valid strings, we note StackRNN has the most novelty, with the least overlap of the strings generated compared to the training data, followed by DNC and NTM. With over 95% of the generated strings being novel across the Generators, the models biased to minimize logP have a higher percentage of novel strings when compared to their unbiased counterparts that presented with a novelty of about 92% across the Generators.

The average length of the strings generated is the highest with StackRNN at 38.83, which is higher than the average length of the strings in the training data set at about 36.15. Strings generated by the NTM are seen to have the least average length (29.14) among the models.

NTM presents with the lowest median SA score of 2.82, followed closely by DNC with 2.95 and StackRNN with a median SA score of 3.08. While being fairly close to each other, these are slightly higher than the median SA score of 2.5 seen with the unbiased models. All the same, the SA score of the molecules generated by the models biased to minimize logP are in the same ballpark as those generated by the unbiased models and not significantly harder to synthesize.

#### Maximizing logP

**Fig 9:**
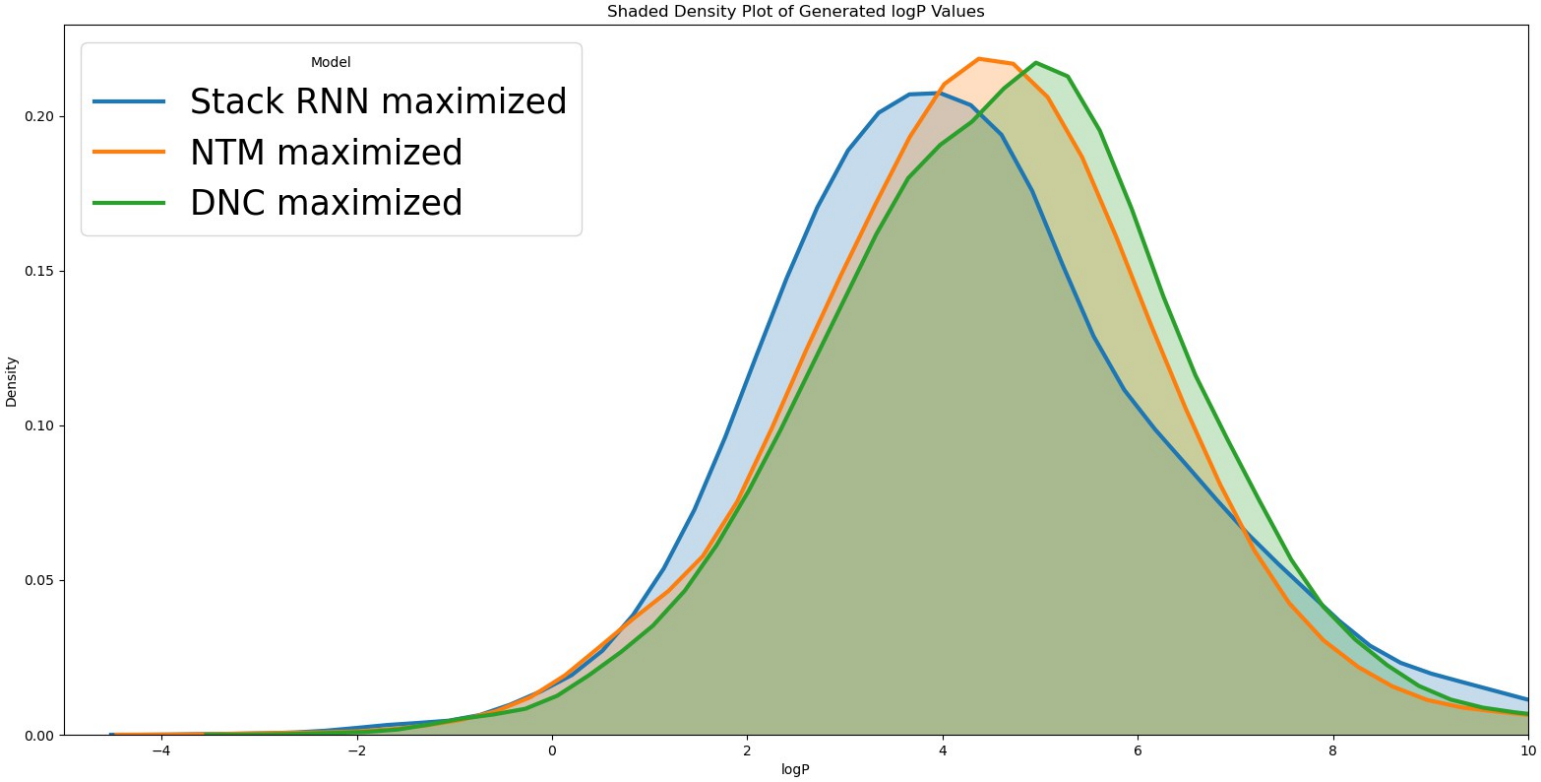
A comparison of the external memory augmented generators biased to maximize logP.

The three Memory Augmented RNN Generator models are biased to generate strings that are valid but with a higher logP value than in the training data. From the density plots in Fig 9, we note the biasing is stronger for the DNC and least for StackRNN. The quartiles of logP and mean logP for the strings generated by three models reported in Table 9 are consistent with this observation.

Of the strings generated by the models, only 90% of the strings generated by StackRNN are valid, whereas 95.5% of the strings generated by NTM and 94.62% of the strings generated by DNC are valid.

Among the strings generated, the novelty is much higher than the unbiased models and models biased to minimize logP. Of the valid strings generated by DNC, about 99.3% of the strings are novel. Even with the StackRNN and NTM, it is noted that over 98% of the strings generated are novel.

Does the biasing and novelty imply the models have learned to generate valid substrings of the training data with the desired property? This is immediately refuted by the average lengths of the molecules generated; as noted from values in Table 9, the average lengths of strings generated by all three biased models are comparable to the average length of strings in training data (viz., 36.15) and the maximized models exceeds the average lengths of the strings generated by their corresponding minimized models. The highest average length for the maximized models is 41.59 which is recorded by StackRNN, followed by DNC with 37.94 and NTM with 36.05.

The median SA score continues to be the lowest for NTM with 2.84, though the median SA score for StackRNN and DNC are not vastly different.

As demonstrated by the results that we have obtained, by employing memory augmented variants of the recurrent neural network, we have seen success in training models to generate 1D enumerations of novel and unique small molecules. The novelty as well as feasibility of these molecules generated using the StackRNN, NTM and DNC is explored and compared. In this comparison of the most optimized model of each kind, the NTM and DNC, perform better than the StackRNN. Further evidence for this is presented in Section 5.4 of the S5 File.

It is interesting to note that the GRU model was comparable to the memory augmented architectures in the task of minimizing logP and the performance of both the LSTM and GRU were comparable to the memory augmented models in the task of maximizing logP. This can be attributed to the ineffectiveness of augmented memory beyond a particular limit, as described through the lower computational (LSTM) units experiments (see Tables 5–8). Among the baseline models, Vanilla RNN did not actually produce novel strings and presented with a high overlap with the training dataset.

#### Post biasing of the generative model

**Fig 10:**
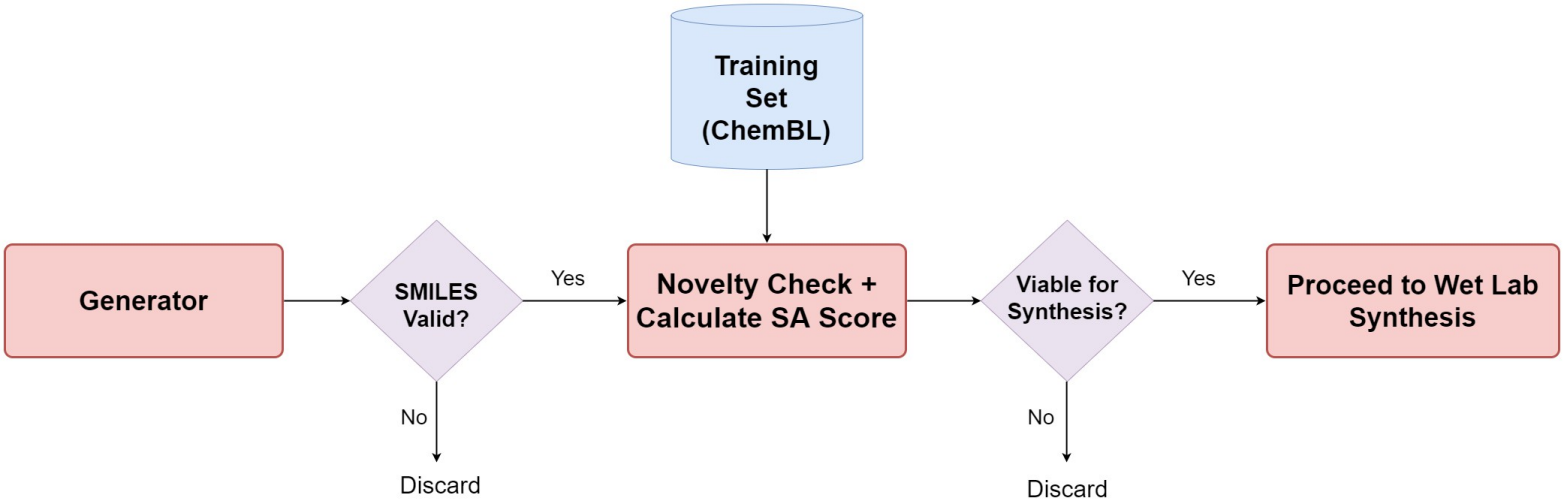
Post training and biasing of generative model.

In summary, the setup comprises a Generator that generates SMILES strings, biased towards one or more properties of interest. These are checked for validity. The novelty and viability through computation of the synthetic accessibility (SA) score are also computed. (Strings that are not valid are immediately discarded as depicted through the schematic diagram in Fig 10).

If the SMILES string representation of a small molecule passes these tests, a 2D structural representation with the predicted properties of interest is output (see Section 4 of the S5 File). These can be further evaluated for feasibility of synthesis in the wet lab and relevant processing is taken up.

## Conclusions

We have described the design of three RNN-based models augmented with external memory: StackRNN, NTM and DNC, to generate SMILES strings that represent small molecules. These have been successfully biased towards logP using a Policy Gradient approach. Based on the results we have obtained, we infer that the reinforcement learning framework is indeed successful in that the libraries of biased strings have a distribution that is consistently shifted in the direction of the biasing of the desired property. In particular, the DNC marginally outperforms the NTM and StackRNN, while the NTM, in turn, outperforms the StackRNN in the extent of biasing exhibited by the strings generated.

A study of the strings generated confirms the models have indeed learnt to generate SMILES string representations of valid small molecules whose average lengths compare well with the average length of the strings presented to the models in the training phase. Further, the output of the Generative models shows a high degree of validity and novelty and feasible synthetic accessibility scores with and without biasing. These measures are seen to be comparable across the three memory augmented Generator models, with the output from the NTM presenting generally higher validity scores and lower SA scores in a majority of the cases.

Results for biasing based on the number of Benzene Rings is reported in Section 3 of the S5 File and are consistent with the foregoing findings. Further, in Section 5 of the S4 File, we have included additional metrics [44, 45] for evaluation of performance of the proposed Generative models.

From the results it is interesting to note that the performance of the LSTM is comparable to that of the memory augmented recurrent neural networks. LSTM performed well in tasks such as maximizing the value of logP and minimizing the number of Benzene rings in the generated molecules, while the Vanilla RNN did not perform well in any of the tasks. In particular, Vanilla RNN yielded a low valid percentage and a low average string length for the maximization tasks.

As demonstrated by the experiments to generate small molecules using progressively larger computational (LSTM) units, the advantage of an external memory unit starts to diminish beyond a certain threshold (128 computational units). Under limited memory constraints, the memory augmented RNNs are likely to produce more favorable results than their vanilla counterparts.

While logP and the number of Benzene rings in the output strings have been used as placeholder properties to demonstrate the ability of the framework to bias the Generators, the Generative models can be biased towards one or more desirable properties or a specific target in actual application.

### Reproducible research

In the spirit of reproducible research, we have made available the source code for all three Generators and SMILES strings generated by all three Generators under all the experimental conditions reported in this paper and in the Supplementary Material (i.e., unbiased Generators and Generators biased for optimizing logP and for optimizing the number of Benzene Rings).

## Supporting information

S1 File Contains details of the organization of the folders of the research repository containing the data, etc., and details of the programming environment. (ZIP)

S2 File Contains the strings used for training and evaluating the various models. (ZIP)

S3 File Has the code for the various generators, the predictor and the reinforcement learning framework to facilitate reproducing the results reported in this study. (ZIP)

S4 File Has the output for the various evaluation metrics, the 2D structural representation and the actual strings generated for the various models (unbaised and biased) reported in this work. (ZIP)

S5 File Contains additional information on the data, details of the models and other evaluation metrics. (PDF)
