# Supplementary material for: Memory augmented recurrent neural networks for de-novo drug design
Source: PLoS One. 2022 Jun 23;17(6):e0269461. doi: 10.1371/journal.pone.0269461 (PMC9223405; doi:10.1371/journal.pone.0269461)
Supplement: S5 File — (PDF) [file pone.0269461.s005.pdf]

Memory augmented recurrent neural networks for de-novo drug design

Naveen Suresh, Neelesh C.A., Srikumar Subramanian and Gowri Srinivasa

Supplementary Material

Table of Contents

1. Dataset profile 4
1.1 Bar plot of atomic types in the dataset 4
1.2 Numerical properties of molecules in dataset 5
1.2.1 Dataset for the generator 5
1.2.1.1 Table of properties 5
1.2.1.2 Three-dimensional scatterplot of Log P values, number of benzene rings and molecular weight for the dataset. 5
1.2.1.3 Data sliced by the number of benzene rings 5
1.2.2 Dataset for the predictor 6
1.2.2.1 Complete dataset 6
1.2.2.1 Sample1 6
1.2.2.2 Sample 2 7
1.2.2.3 Sample 3 **Error! Bookmark not defined.**
1.2.2.4 Sample 4 **Error! Bookmark not defined.**
1.3 Salts in dataset 8

2. Tuning of Hyperparameters 9
2.1 Stack RNN 9
2.2 NTM 9
2.3 DNC 12
2.4 Jaccard index for unbiased generators 13

3. Results for biasing on number of Benzene rings 14
3.1 StackRNN 14
3.2 NTM 15
3.3 DNC 16
3.4 Unbiased Results 17
3.5 Minimization Results 18
3.6 Maximization Results 20
3.7 Predictor Results 21
3.8 Common string percentage over entire data 21

4. Average length of the SMILES strings generated 21
3.1 Unbiased models 21

4.1.1 Stack RNN 21
4.1.2 NTM 22
4.1.3 DNC 22
4.2 Models biased for logP 22
4.2.1 Stack RNN maximized 22
4.2.2 Stack RNN minimized 22
4.2.3 NTM maximized 22
4.2.4 NTM minimized 23
4.2.5 DNC maximized 23
4.2.6 DNC minimized 23
4.3 Models biased for the number of Benzene Rings 23
4.2.1 Stack RNN maximized 23
4.2.2 Stack RNN minimized 23
4.2.3 NTM maximized 24
4.2.4 NTM minimized 24
4.2.5 DNC maximized 24
4.2.6 DNC minimized 24
5. Additional Metrics 25
5.1 MOSES Metrics 25
5.1.1 Unbiased models 25
5.1.2 DNC biased towards benzene 26
5.1.3 DNC biased towards Log P 27
5.1.4 NTM biased towards benzene 27
5.1.5 NTM biased towards Log P 27
5.1.6 Stack RNN biased towards benzene 27
5.1.7 Stack RNN biased towards Log P 28
5.1.8 Unbiased models (Wasserstein-1 distance) 28
5.1.9 DNC biased towards benzene (Wasserstein-1 distance) 29
5.1.10 DNC biased towards Log P(Wasserstein-1 distance) 29
5.1.11 NTM biased towards benzene (Wasserstein-1 distance) **Error! Bookmark not defined.**
5.1.12 NTM biased towards Log P(Wasserstein-1 distance) 30
5.1.13 Stack RNN biased towards benzene (Wasserstein-1 distance) 30
5.1.14 Stack RNN biased towards Log P (Wasserstein-1 distance) 30
5.1.15 Control (Wasserstein-1 distance) 31
5.2 Fréchet ChemNet Distance 31
5.3 Common string percentage with additional data 32
5.3.1 Common string percentage 32
5.3.2 Data Sources 33
5.4 Mean and Std Dev of property values 35
5.4.1 LogP 35

5.4.2 Benzene Rings 35
5.5 Mean and Std Dev of valid percentage 36
5.5.1 Unbiased 36
5.5.2 LogP optimized models 36
5.5.3 Benzene Rings optimized models **Error! Bookmark not defined.**

6. Comparison of models with lower memory 37
6.1 Validity percentage for models with 16 units 38
6.2 Validity percentage for models with 32 units 38
6.3 Validity percentage for models with 64 units 39
6.4 Validity percentage for models with 128 units **Error! Bookmark not defined.**

7. 2-D representation of molecule generated 40

1. Dataset profile

The dataset for the generator was sampled such that molecular weight did not exceed 900 daltons. This was chosen to ensure that the generator was trained only on small molecules. The number of strings for the generator were 788,452. We did not canonicalize strings in the dataset nor remove duplicates. Among the set of 788,452 strings 212,196 molecules were in canonical form. The set of 788,452 strings included 29,656 duplicates.

For the predictor, there may be cases where the input to it may not be a small molecule. E.g. in cases where the generator ends up generating molecules with weight > 900 daltons.
Hence, in order to cover all the possibilities, the sample was 200,000 molecules without any filtering.

In section 5.2.2, it can be seen that the properties of the sample of 200,000 are similar to the complete dataset. Only range varies, but that is expected as that is sensitive to outliers.

1.1 Bar plot of atomic types in the dataset

Number of strings in dataset = 788,452
These atoms were obtained from rdkit.

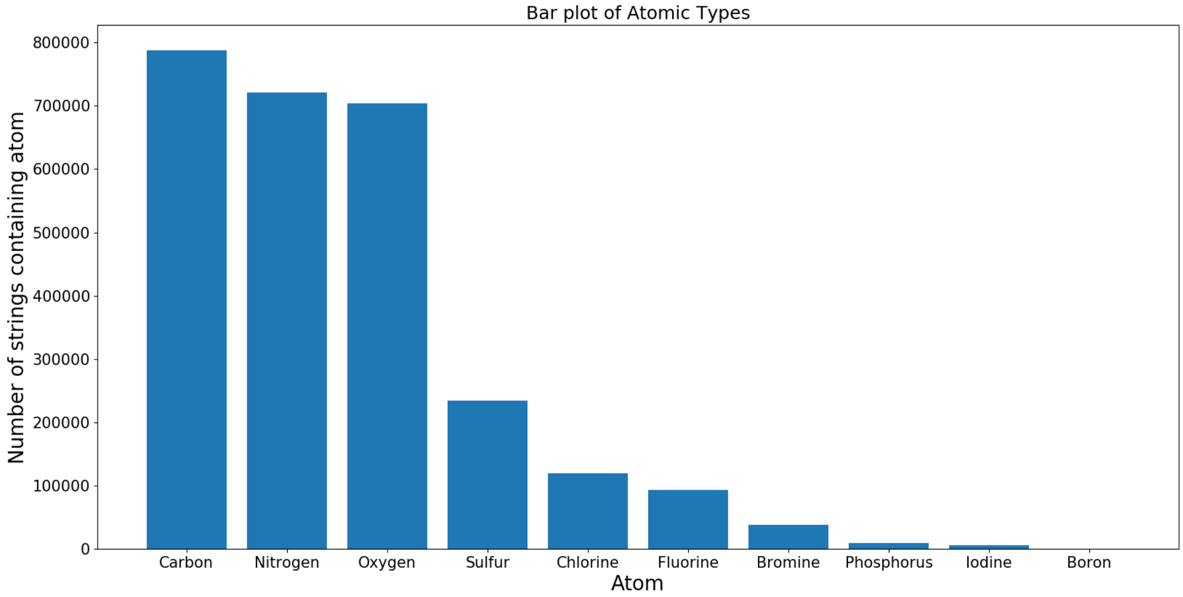

Fig 1. Bar plot of atomic types in the dataset

1.2 Numerical properties of molecules in dataset

1.2.1 Dataset for the generator

1.2.1.1 Table of properties

Property	Q1	Q2	Q3	Mean	Range
Mol Wt	278.173	320 .164	354.138	314.136	798.707
LogP	1.982	2.941	3.858	2.877	20.864
Benzene	1.0	1.0	2.0	1.180	7

Table 1 - Table of properties for the dataset for the generator

1.2.1.2 Three-dimensional scatterplot of Log P values, number of benzene rings and molecular weight for the dataset.

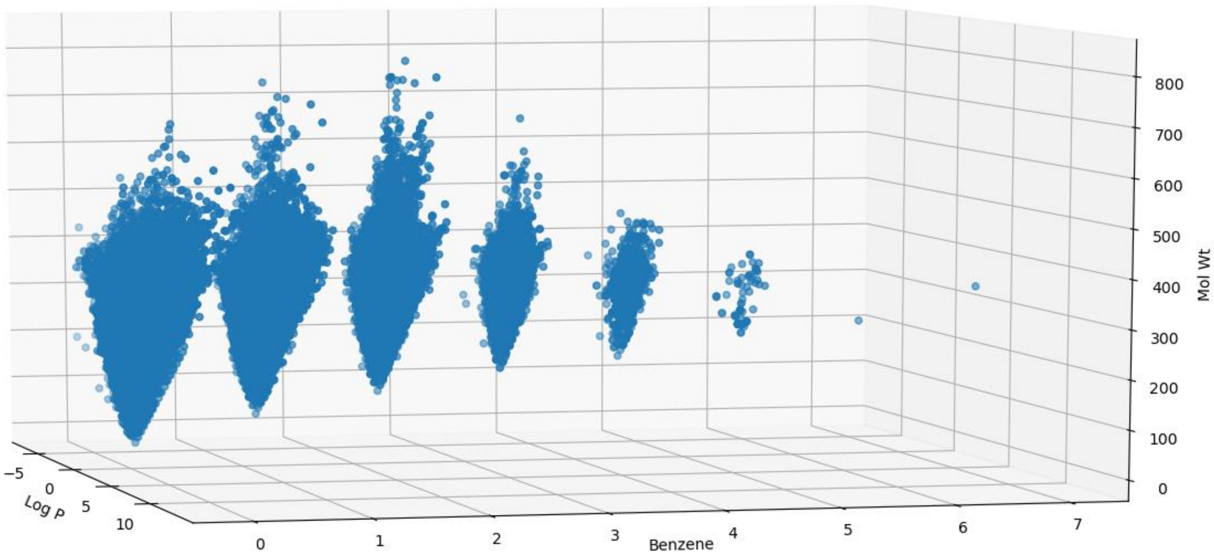

Fig 2. 3-D scatterplot of LogP values, number of benzene rings and mol wt. for dataset

1.2.1.3 Data sliced by the number of benzene rings

Number of benzene	Number of molecules	Minimum LogP	Maximum LogP	Minimum molecular	Maximum Molecular

rings				weight	Weight
0	156936	-7.12	13.74	16.03	691.58
1	361074	-3.72	10.98	78.05	762.86
2	242733	-0.92	10.62	128.06	814.74
3	26549	-0.04	9.1	178.08	696.81
4	1084	1.47	8.77	202.08	496.04
5	75	3.3	8.03	252.09	416.23
6	1	6.33	6.33	276.09	276.09
7	1	6.83	6.83	345.08	345.08

Table 2 - Table of properties for the dataset for the generator sliced by number of benzene rings

From the above graph and table, we can see that most of the molecules used for training the dataset contain 0-2 benzene rings. There is a slight variation for the logP values and molecular weight, with the range of logP values decreasing with the number of benzene rings while the range of molecular weight is shifted upwards. For 3-4 benzene rings, the values are also similar although the range is smaller, probably due to the absence of outliers. For 5-7 benzene rings, the number of molecules is too small to make any meaningful conclusion.

1.2.2 Dataset for the predictor

1.2.2.1 Complete dataset

Property	Q1	Q2	Q3	Mean	Range
Mol Wt	323.19	389.12	463.29	399.93	1442.134
Log P	2.39	3.47	4.6	3.49	35.49
Benzene	1.00	2.00	2.00	1.52	9.00

Table 3 - Table of properties for the dataset for the predictor

1.2.2.1 Sample1

Property	Q1	Q2	Q3	Mean	Range

Mol Wt	323.10	388.18	463.23	399.52	1,231.94
Log P	2.38	3.47	4.59	3.49	28.25
Benzene	1.00	1.00	2.00	1.51	9.00

Table 4 - Table of properties for one sample of the dataset for the predictor

1.2.2.2 Sample 2

Property	Q1	Q2	Q3	Mean	Range
Mol Wt	324.10	389.15	463.22	399.87	1,292.80
Log P	2.39	3.47	4.60	3.50	28.25
Benzene	1.00	2.00	2.00	1.52	8.00

Table 5 - Table of properties for one sample of the dataset for the predictor

1.2.2.3 Sample 3

Property	Q1	Q2	Q3	Mean	Range
Mol Wt	323.15	388.19	463.19	399.62	1,293.79
Log P	2.39	3.47	4.59	3.49	27.23
Benzene	1.00	1.00	2.00	1.51	9.00

Table 6 - Table of properties for one sample of the dataset for the predictor

1.2.2.4 Sample 4

Property	Q1	Q2	Q3	Mean	Range
Mol Wt	323.88	389.11	463.17	399.82	1,191.67
Log P	2.38	3.47	4.60	3.49	30.98
Benzene	1.00	2.00	2.00	1.51	9.00

Table 7 - Table of properties for one sample of the dataset for the predictor

1.3 Salts in dataset

In the dataset of 788,452 molecules, 15 molecules contained salts. These molecules were not de-salted. Among the molecules generated by the model; only the DNC model biased towards minimizing the value of logP contained 1 salted molecule out of the 4000 molecules it had generated. Molecules generated by all other models did not contain any salt.

Salts present (unique salts)

CC(O)=O
OP(O)(O)=O
OS(O)(=O)=O
OC(=O)C(O)=O
CS(O)(=O)=O
OC(=O)C(F)(F)F
OC(=O)C=CC(O)=O
F[P-](F)(F)(F)(F)F
C1CCC(CC1)NC1CCCCC1
OC(C(O)C(O)=O)C(O)=O
Cc1ccc(cc1)S(O)(=O)=O

2. Tuning of Hyperparameters

NTM was the first generator which was trained. Hence, once we determined its hyper parameters, the same was applied to the stack RNN and DNC to ensure a fair comparison.

2.1 Stack RNN

(For results mentioned in Tables 3 and 4 of the main paper)

**Parameter**	**Value**
embedding_length	1024
num_layers	1
num_controller_layers	num_layers
num_units	1024
num_controller_units	num_units
stack_width	1024
stack_depth	200
learning_rate	0.001
max_grad_norm	50
batch_size	26

Table 8 - Hyperparameters for the stack RNN

As mentioned earlier, the hyperparameters for stack RNN are similar to the NTM. The stack_width corresponds to the memory_size and the stack_depth corresponds to the num_memory_locations.
The implementation of stackRNN required more memory, hence a smaller batch_size was used. The read/write heads are missing as the stackRNN does not have that capability.

2.2 NTM

(For results mentioned in Tables 3 and 4 of the main paper)

The memory size was chosen to be a power of 2 to keep in line with computer architecture. The embedding length is the same as the memory size so that there’s no wastage/overuse of

memory. The number of units is also the same as the memory size, so that each unit can handle one cell.

**Parameter**	**Value**
embedding_length	1024
num_layers	1
num_controller_layers	num_layers
num_units	1024
num_controller_units	num_units
num_memory_locations	200
memory_size	1024
num_read_heads	1
num_write_heads	1
conv_shift_range	1
clip_value	20
clip_controller_output_to_value	clip_value
init_mode	constant

learning_rate	0.001
max_grad_norm	50
Batch_size	38
num_bits_per_vector	embedding length

Table 9 - Hyperparameters for the NTM

To determine the embedding length/memory size, which was also used as the value for the number of units, we tried 5 different values which are mentioned in table 3. The batch sizes were inversely proportional to the embedding length. For an embedding length of 512 and 1500, they are markedly different from the other 3. 768, 1024 and 1280 and all fairly close, with a range of 1.5% on valid strings, and 0.5% on common strings. We went with 1024 as it can be considered the median, and we did not have any strong bias towards either valid string or common string %.

**Embedding Length**	**Valid String %**	**Common String %**
512	94.3	7.51
768	96.26	9.77
**1024**	**95.33**	**9.58**
1280	94.76	9.28
1500	89.35	3.01

Table 10 - Valid String and Common String % for various embedding lengths of the NTM

The number of memory locations is greater than the length of the SMILE, so that there is enough memory for the whole SMILE.
The number of controllers layers/read head/write heads are 1, as any more than that led to worse results.
We generated strings at 11000th and 13000th iterations when training a model with 2 read heads/write heads/controller layers and the percentage of valid strings were 77 % and 55% respectively which is a very steep downward decline. When considering that there were models which generated valid strings at greater than 90%, the results here did not seem worth pursuing.

This also keeps it in line with the stack RNN, where these are not configurable parameters.

The conv_shift_range, clip_value, learning_rate and max_grad_norm are the same as from https://arxiv.org/abs/1807.08518 which was the NTM paper from where we took our implementation.
The init_mode is constant which gave the best results for the above mentioned paper.
The batch size chosen was the maximum value which would fit in our GPU configuration considering the other memory requirements(number of memory units, embedding length etc)

2.3 DNC

(For results mentioned in Table 3 and 4 of the main paper)

The DNC is more similar to the NTM compared to the stack RNN, hence the parameters are essentially the same.

Parameter	Value
embedding_length	1024
num_layers	1
num_controller_layers	num_layers
num_units	1024
num_controller_units	num_units
num_memory_locations	200
memory_size	1024
num_read_heads	1

num_write_heads	1
conv_shift_range	1
clip_value	20
clip_controller_output_to_value	clip_value
init_mode	constant
learning_rate	0.001
max_grad_norm	50
Batch_size	26
num_bits_per_vector	embedding length

Table 11 - Hyperparameters for the DNC

2.4 Jaccard index for unbiased generators

**Number of Iterations**	**StackRNN**	**NTM**	**DNC**
5000	9.53	13.8	7.2
10000	11.8	17.3	9.3
15000	15.0	21.2	10.4
20000	17.1	21.2	13.7
25000	22.8	26.2	19.2

30000	23.0	34.2	17.4
35000	19.5	31.2	16.4
40000	23.9	38.6	18.4
45000	20.4	39.1	23.1

Table 12 - Jaccard index * 10^5 for strings generated by each model at a particular iteration

The Jaccard index for all of the models more or less increased with the number of iterations. This is expected as the more training data the model sees, the more similar its output becomes. There is some variation, e.g. for stackRNN it decreases from 30000 to 35000, and for DNC it decreases from 25000 to 35000, but these can be considered a variation in training.

At the end of 45000 iterations, the NTM had the highest jaccard index, with the stack RNN the least. This meant that the NTM produced more molecules that were present in the training set. However, it should be noted that there was no explicit optimization done for reducing the amount of common strings between training data and what the model generates.

3. Results for biasing on number of Benzene rings

3.1 StackRNN

	Q1	Q2	Q3	Mean	Valid String %	Common String %	SAS Median
Minimized Benzene ring	0	0	1	0.39	94.31	1.39	3.44
Unbiased Benzene ring	1	1	2	1.16	93.12	4.86	2.56
Maximized Benzene ring	1	2	3	2.01	81.38	0	3.4

Table 13 - Comparison of biased stack RNN models

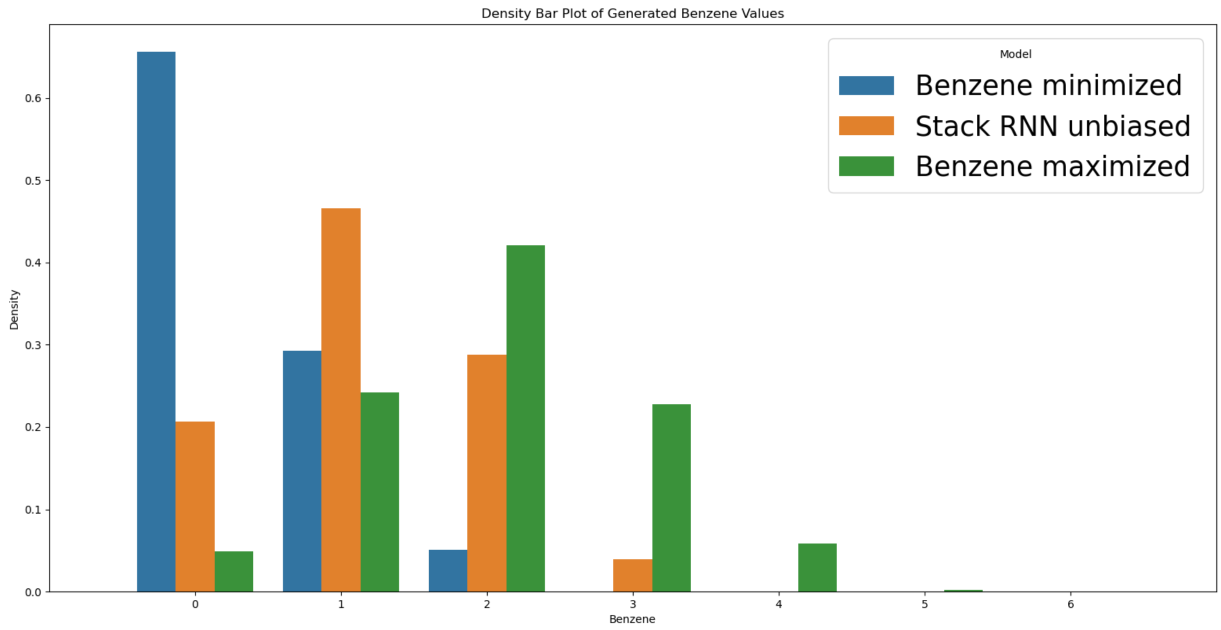

Fig 3. Comparison of biased Stack RNN models

As seen in the above data, when considering the number of benzene rings as the property to be biased, the StackRNN distribution shifts from the unbiased distribution towards the maximum in the maximized model and towards the minimum in the minimized model. It is also worth noting that the multi-modal appearance of the graph is because of the discrete nature of the predicted value.

3.2 NTM

	Q1	Q2	Q3	Mean	Valid String %	Common String %	SAS Median
Minimized Benzene ring	0	0	0	0.15	89.26	1.06	2.66
Unbiased Benzene ring	1	1	2	1.24	94.71	8.2	2.52
Maximized Benzene ring	2	2	3	2.26	96.42	5.13	2.01

Table 14 - Comparison of biased NTM models

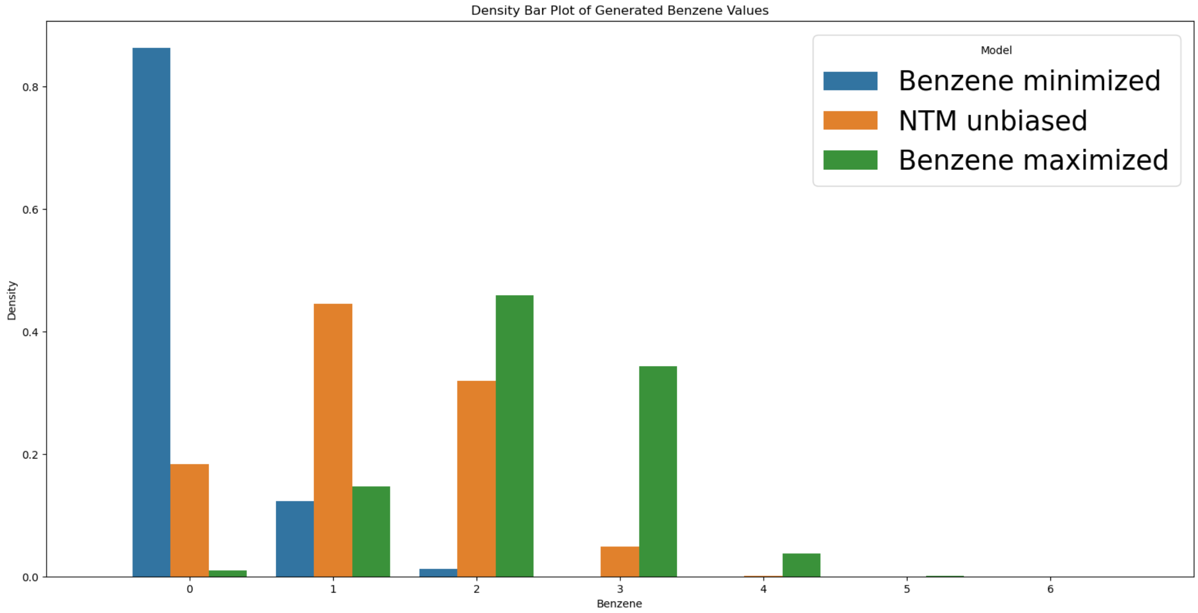

Fig 4. Comparison of biased NTM models

Similar to the case of the stackRNN, the NTM also displays a shift from the unbiased distribution towards the maximum in the maximized model and towards the minimum in the minimized model.

3.3 DNC

	Q1	Q2	Q3	Mean	Valid String %	Common String %	SAS Median
Minimized Benzene ring	0	0	0	0.14	93.15	1.65	3.17
Unbiased Benzene ring	1	1	2	1.11	94.77	7.08	2.53
Maximized Benzene ring	2	3	4	2.68	96.54	1.04	2.75

Table 15 - Comparison of biased DNC models

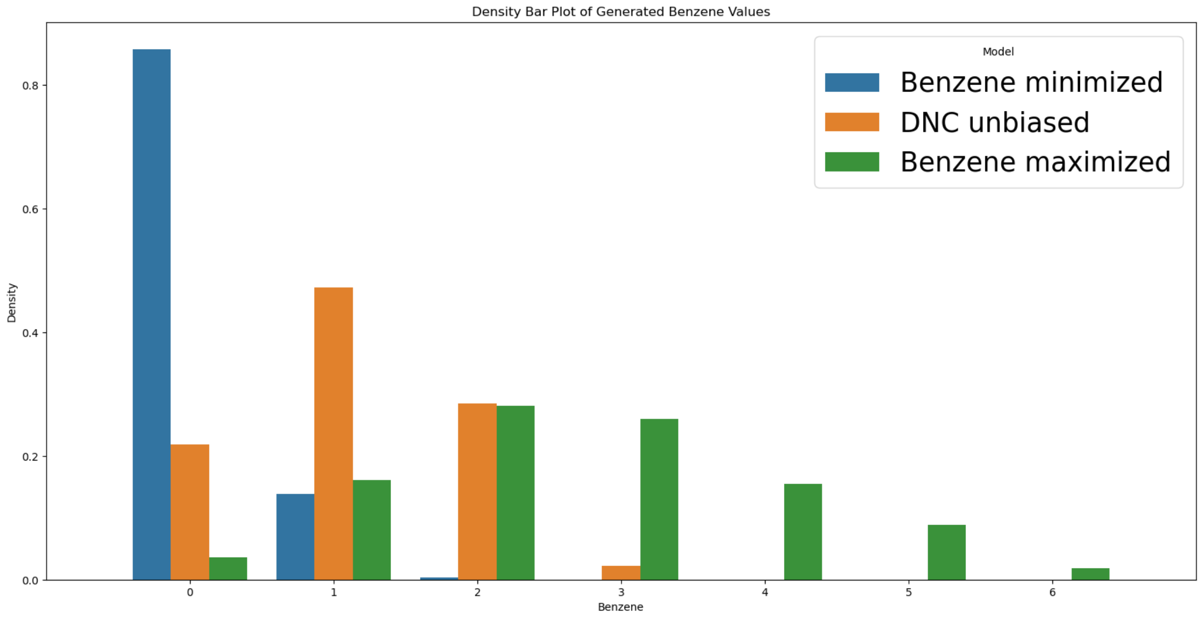

Fig 5. Comparison of biased DNC models

Much like the previous two generators, the DNC also displays a shift from the unbiased distribution towards the maximum in the maximized model and towards the minimum in the minimized model.

3.4 Unbiased Results

		Q1	Q2	Q3	Mean	Valid String %	Common String %	SAS Median
Memory Augmented Models	StackRNN	1	1	2	1.16	93.12	4.85	2.56
	NTM	1	1	2	1.24	94.71	8.2	2.52
	DNC	1	1	2	1.11	94.77	7.07	2.53
Baseline Models	GRU	1	1	2	1.23	82.43	2.12	2.58
	LSTM	1	1	2	1.14	92.58	5.16	2.53
	Simple RNN	1	1	2	1.11	31.23	3.6	2.83

Table 16 - Comparison of unbiased models

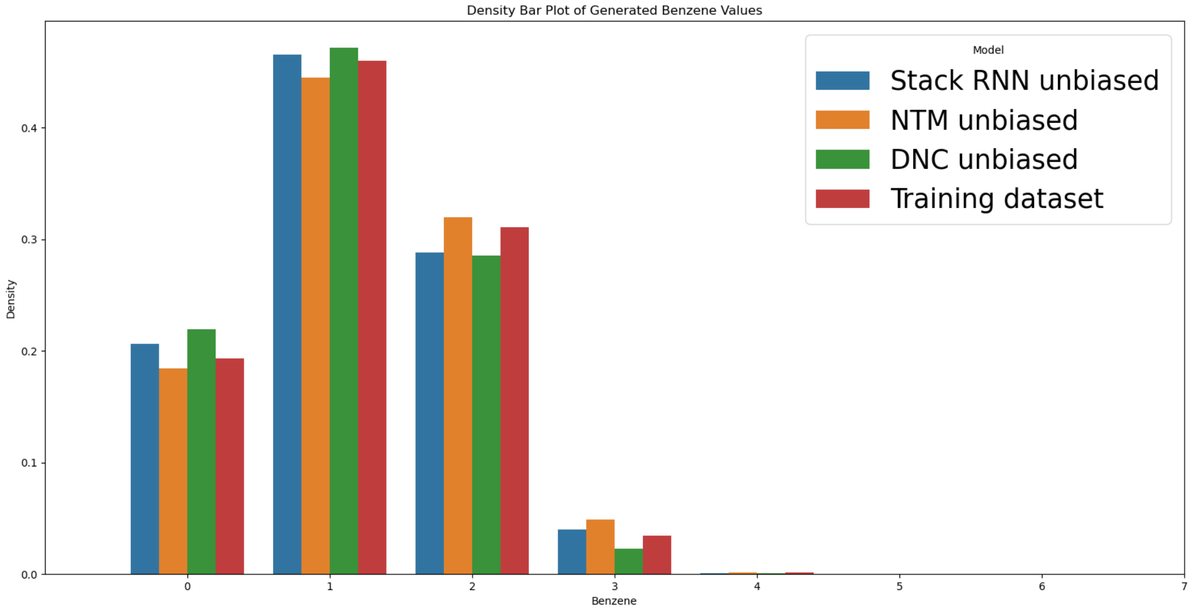

Fig 6. Comparison of unbiased external memory augmented Generators

The distributions of all three memory augmented neural networks, before biasing, are nearly identical. The quartiles are identical with negligible differences in mean. Similar results are observed with the baseline models as well, with the exception of the vanilla RNN, which struggled to generate valid strings. This is the same as the models used in the unbiased LogP experiments (no biasing done).

The training dataset is also very similar to the unbiased models.

3.5 Minimization Results

		Q1	Q2	Q3	Mean	Valid String %	Common String %	SAS Median
Memory Augmented Models	StackRNN	0	0	1	0.39	94.31	1.39	3.44
	NTM	0	0	0	0.15	89.26	1.06	2.66
	DNC	0	0	0	0.14	93.15	1.65	3.17
Baseline Models	GRU	1	1	1	0.79	91.83	0.82	2.75
	LSTM	0	0	1	0.5	89.65	1.59	3.02
	Vanilla RNN	1	1	1	0.991	98.95	0.007	1.90

Table 17 - Comparison of minimized models

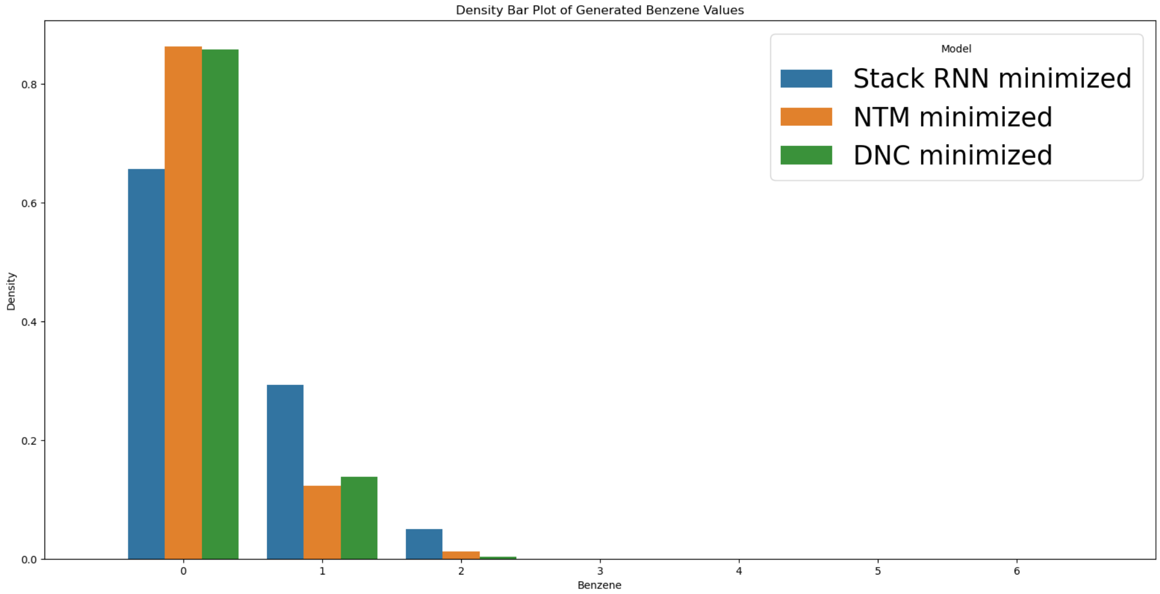

Fig 7. Comparison of minimized external memory augmented Generators

The minimized set of generators have all shifted their distribution from the unbiased models towards a lower number of benzene rings. It is evident that the biasing was more effective for the NTM and the DNC than it was for the StackRNN. Q3 for the StackRNN was 1 while it was 0 for the NTM and DNC. The DNC and NTM have comparable distributions, but the DNC was able to generate a higher set of valid strings (93.15% vs 89.26%). The NTM did produce molecules with lower synthetic accessibility strings (NTM: 2.66 vs DNC: 3.17 vs StackRNN: 3.44), although all of them produced a large number of strings lesser than the heuristic value of 6 (the approximate cutoff for molecules that are difficult to synthesize).

The baseline models (particularly the GRU and the Vanilla RNN) were unable to bias their respective distributions towards minimizing benzene rings effectively. This is embodied in the nearly unaffected distributions in comparison to the unbiased models. The Vanilla RNN was also observed to overfit, with 42 unique strings out of 4000 generated strings. The LSTM was able to achieve some degree of biasing, but the memory-augmented RNNs (specifically the NTM and the DNC) were able to outperform the LSTM.

3.6 Maximization Results

		Q1	Q2	Q3	Mean	Valid String %	Common String %	SAS Mean
Memory Augmented Models	StackRNN	1	2	3	2.01	81.38	0	3.4
	NTM	2	2	3	2.26	96.42	5.13	2.01
	DNC	2	3	4	2.68	96.54	1.04	2.75
Baseline Models	GRU	1	2	2	1.78	91	3.02	2.26
	LSTM	1	2	2	1.76	91.2	11.6	2.03
	Vanilla RNN	2	2	2	1.91	96	10.6	1.33

Table 18 - Comparison of Maximized models

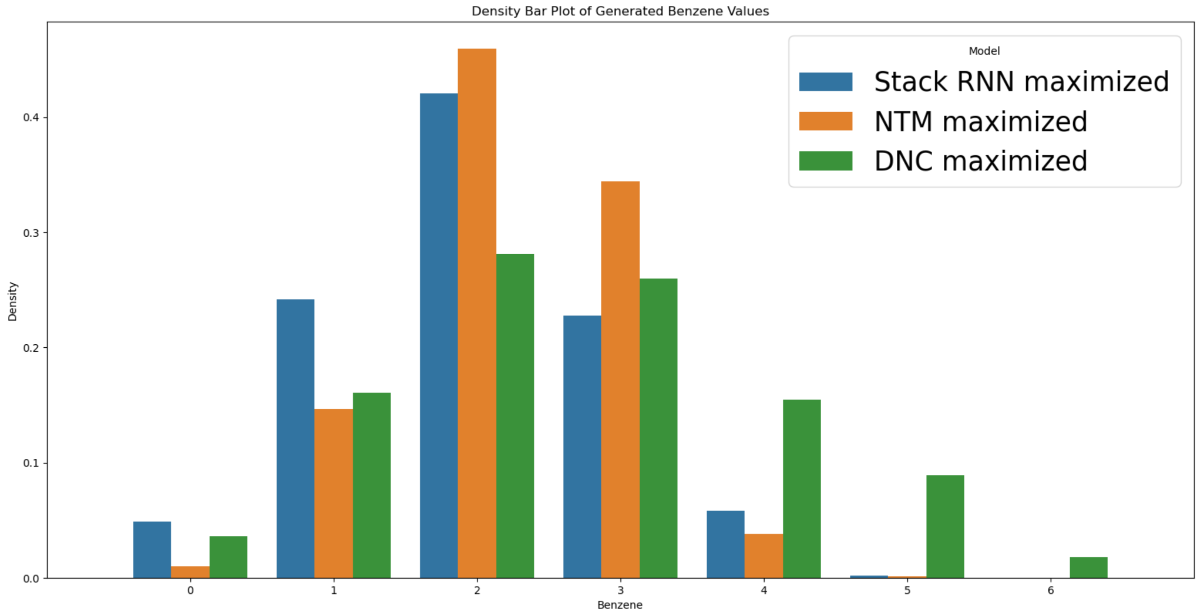

Fig 8. Comparison of maximized external memory augmented Generators

The maximized set of generators have all shifted their distribution from the unbiased models towards a higher number of benzene rings. There is a discernible difference between the three generated distributions, with the NTM outperforming the StackRNN and the DNC outperforming both models. The extent of biasing is the greatest for the DNC by a significant margin, and is the only model to have 25% of strings generated to have more than 4 benzene rings. The DNC and NTM also significantly outperform the StackRNN in terms of generation of valid strings, while maintaining an insignificant overlap with the training set. The median of the synthetic accessibility scores are also well within the permissible limit of 6.

Similar to the case of minimization of benzene rings, the baseline models were unable to bias their respective distributions towards maximizing benzene rings effectively. While the Vanilla RNN seemed to bias more effectively than the GRU and LSTM models, the Vanilla RNN could only generate 218 unique strings out of 4000 generated. This suggests that the model overfit, and hence does not generate the novelty as captured by the common string percentage. The GRU and LST models biased far less effectively than the memory augmented models.

3.7 Predictor Results

The character level CNN achieved an MSE of **0.039** for the prediction of the number of Benzene rings in the SMILES representation of the molecule.

3.8 Common string percentage over entire data

It should be noted that the training dataset used was only half of what was available. When the common string percentage was calculated with the fully available dataset, the common string percentages increased by around 0.016, 0.04 and 0.02 for the stack RNN, NTM and DNC respectively. An increase is to be expected, but it is fairly small.
For the biased models, the common string % is exactly the same when either half of the dataset is used, or the full dataset. The same is true for log P as well.

4. Average length of the SMILES strings generated

It can be seen in the tables below that the biasing has an effect on the length of the strings generated. The length is lower when trying to minimize the value of logP and when trying to minimize the number of benzene rings in the molecule. Whereas when trying to bias towards a higher value of logP and bias towards higher number of benzene rings in the molecule, longer strings are generated by the model.

3.1 Unbiased models

4.1.1 Stack RNN

q1	q2	q3	mean	min	max
31.0	37.0	42.0	36.005	3	61

Table 19 - Distribution of string lengths for unbiased stack RNN

4.1.2 NTM

q1	q2	q3	mean	min	max
32.0	37.0	41.0	36.06	4	56

Table 20 - Distribution of string lengths for unbiased NTM

4.1.3 DNC

q1	q2	q3	mean	min	max
31.0	36.0	41.0	35.676	5	59

Table 21 - Distribution of string lengths for unbiased DNC

4.2 Models biased for logP

4.2.1 Stack RNN maximized

q1	q2	q3	mean	min	max
33.0	40.0	45.0	41.591	10	100

Table 22 - Distribution of string lengths for stack RNN biased towards maximizing log P

4.2.2 Stack RNN minimized

q1	q2	q3	mean	min	max
34.0	40.0	45.0	38.835	2	71

Table 23 - Distribution of string lengths for stack RNN biased towards minimizing log P

4.2.3 NTM maximized

q1	q2	q3	mean	min	max
33.0	38.0	43.0	36.057	10	100

Table 24 - Distribution of string lengths for NTM biased towards maximizing log P

4.2.4 NTM minimized

q1	q2	q3	mean	min	max
23.0	30.0	36.0	29.141	2	52

Table 25 - Distribution of string lengths for NTM biased towards minimizing log P

4.2.5 DNC maximized

q1	q2	q3	mean	min	max
32.0	37.0	43.0	37.945	4	100

Table 26 - Distribution of string lengths for DNC biased towards maximizing log P

4.2.6 DNC minimized

q1	q2	q3	mean	min	max
30.0	36.0	41.0	35.045	5	64

Table 27 - Distribution of string lengths for DNC biased towards minimizing log P

4.3 Models biased for the number of Benzene Rings

4.2.1 Stack RNN maximized

q1	q2	q3	mean	min	max
41.0	64.5	81.0	65.407	14	100

Table 28 - Distribution of string lengths for stack RNN biased towards maximizing number of benzene rings

4.2.2 Stack RNN minimized

q1	q2	q3	mean	min	max
31.0	39.0	46.0	38.262	3	69

Table 29 - Distribution of string lengths for stack RNN biased towards minimizing number of benzene rings

4.2.3 NTM maximized

q1	q2	q3	mean	min	max
30.0	36.0	43.0	36.524	4	59

Table 30 - Distribution of string lengths for NTM biased towards maximizing number of benzene rings

4.2.4 NTM minimized

q1	q2	q3	mean	min	max
32.0	39.0	45.0	38.975	9	100

Table 31 - Distribution of string lengths for NTM biased towards minimizing number of benzene rings

4.2.5 DNC maximized

q1	q2	q3	mean	min	max
33.0	42.0	49.0	41.568	9	73

Table 32 - Distribution of string lengths for DNC biased towards maximizing number of benzene rings

4.2.6 DNC minimized

q1	q2	q3	mean	min	max
27.0	33.0	38.0	32.747	9	59

Table 33 - Distribution of string lengths for DNC biased towards minimizing number of benzene rings

5. Additional Metrics

The metrics are presented for all of our models. The 3 baseline models, 3 unbiased memory augmented networks, and 12 biased memory augmented networks (maximized/minimized log P/benzene)

5.1 MOSES Metrics

The following tables have metrics from MOSES.

30,000 strings were generated for each model as recommended by moses. The training set was chosen such that the average string length was around 37. The test/reference set in this case has an average string length of 48.
We did not have a scaffold test set as that was not the focus of the project.
There was no filtering done on our dataset, hence the filter values are not reported either.

It should be noted that while training, the only metric that was optimized was validity for the unbiased models. While the biased models were additionally optimized towards their target property.

The following metrics are reported

1. Frag/Test - Measures similarity of fragments between the test and generated set.
2. IntDiv/IntDiv2 - Measures diversity of generated test set.
3. Novelty - Measures proportion of generated molecules that are not present in the training set.
4. SNN - Measures similarity between fingerprints of a molecule in the generated set and it's nearest neighbour in the test set.
5. Scaf/Test - Measures similarity of Bemis–Murcko scaffolds between the test and generated set.
6. Unique - Measures uniqueness of molecules generated.
7. Valid - Measures percentage of valid molecules. These have proper valence, brackets that are closed etc.

There are Wasserstein-1 distances reported for molecular properties such as lipophilicity (logP), Synthetic Accessibility (SA), Quantitative Estimation of Drug-likeness (QED) and molecular weight.

5.1.1 Unbiased models

Model	Frag /Test	IntDiv	IntDiv2	Novelty	SNN /Test	Scaf /Test	unique @1000	unique@ 10000	valid
Stack RNN	0.992	0.882	0.876	0.983	0.432	0.487	1	0.999	0.93
NTM	0.994	0.883	0.876	0.975	0.447	0.531	1	0.998	0.95
DNC	0.991	0.883	0.876	0.98	0.444	0.51	1	0.999	0.945
GRU	0.986	0.884	0.877	0.988	0.408	0.447	1	0.998	0.824
LSTM	0.993	0.882	0.875	0.984	0.434	0.489	1	0.999	0.934
Vanilla	0.96	0.883	0.875	0.986	0.377	0.348	1	0.982	0.322

Table 34 - MOSES metrics for unbiased models

The fragment similarity is good for all the unbiased models, with values nearing 1.
The internal diversity is also good, with values around 0.87-0.88.
The novelty is high, with values around 0.98.
For the above metrics, there was not really too much variation across models.

The SNN scores are a bit low, with the highest being 0.447.
The scaffold scores are quite low, with the highest being 0.531.
A control was run as seen in table 6.1.15 which may explain these values.
It can be seen that the vanilla RNN has the worst values for these 2 metrics, followed by the GRU.
The other 4 models are fairly similar in value, however, the NTM and DNC are marginally higher than the stack RNN and DNC.

The uniqueness values are high for all models, with the vanilla RNN havign a marginally lower uniqueness value for 10000 molecules.

For validity, there is a reasonable amount of difference. The vanilla RNN has a validity of only 0.322, while the next highest, which is the GRU has a validity of 0.824.
The other 4 models have validities above 0.93, with the NTM being the highest at 0.95.

5.1.2 DNC biased towards benzene

Model	Frag /Test	IntDiv	IntDiv2	Novelty	SNN /Test	Scaf /Test	unique @1000	unique @10000	valid
Maximized	0.469	0.804	0.786	0.996	0.358	0.06	0.893	0.785	0.967
Minimized	0.823	0.888	0.88	0.993	0.346	0.053	0.995	0.974	0.926
Unbiased	0.991	0.883	0.876	0.98	0.444	0.51	1	0.999	0.945

Table 35 - MOSES metrics for DNC biased towards number of benzene rings

5.1.3 DNC biased towards Log P

Model	Frag /Test	IntDiv	IntDiv2	Novelty	SNN /Test	Scaf /Test	unique @1000	unique @10000	valid
Maximized	0.727	0.852	0.842	0.997	0.368	0.193	0.998	0.993	0.949
Minimized	0.878	0.885	0.877	0.991	0.375	0.126	0.998	0.993	0.922
Unbiased	0.991	0.883	0.876	0.98	0.444	0.51	1	0.999	0.945

Table 36 - MOSES metrics for DNC biased towards Log P

5.1.4 NTM biased towards benzene

Model	Frag /Test	IntDiv	IntDiv2	Novelty	SNN /Test	Scaf /Test	unique @1000	unique@10000	valid
Maximized	0.787	0.823	0.809	0.987	0.46	0.137	0.925	0.789	0.96
Minimized	0.766	0.801	0.78	0.995	0.441	0.157	0.985	0.954	0.898
Unbiased	0.994	0.883	0.876	0.975	0.447	0.531	1	0.998	0.95

Table 37 - MOSES metrics for NTM biased towards number of benzene rings

5.1.5 NTM biased towards Log P

Model	Frag /Test	IntDiv	IntDiv2	Novelty	SNN /Test	Scaf /Test	unique @1000	unique@ 10000	valid
Maximized	0.795	0.847	0.837	0.997	0.392	0.221	0.989	0.95	0.951
Minimized	0.88	0.9	0.888	0.985	0.372	0.27	0.975	0.927	0.958
Unbiased	0.994	0.883	0.876	0.975	0.447	0.531	1	0.998	0.95

Table 38 - MOSES metrics for NTM biased towards Log P

5.1.6 Stack RNN biased towards benzene

Model	Frag /Test	IntDiv	IntDiv2	Novelty	SNN /Test	Scaf /Test	unique @1000	unique@ 10000	valid

Maximized	0.84	0.836	0.831	0.998	0.35	0.198	0.998	0.995	0.878
Minimized	0.739	0.865	0.851	0.995	0.365	0.038	0.992	0.965	0.935
Unbiased	0.992	0.882	0.876	0.983	0.432	0.487	1	0.999	0.93

Table 39 - MOSES metrics for Stack RNN biased towards number of benzene rings

5.1.7 Stack RNN biased towards Log P

Model	Frag /Test	IntDiv	IntDiv2	Novelty	SNN /Test	Scaf /Test	unique @1000	unique@ 10000	valid
Maximized	0.925	0.881	0.872	0.994	0.387	0.381	1	0.998	0.951
Minimized	0.869	0.862	0.853	0.994	0.373	0.13	0.997	0.986	0.968
Unbiased	0.992	0.882	0.876	0.983	0.432	0.487	1	0.999	0.93

Table 40 - MOSES metrics for Stack RNN biased towards Log P

From the above tables for the biased models, we can establish the following trends.
The fragment similarity, SNN, and scaffold similarity lower for the biased models. This means that the biased models are producing molecules which are farther away from the dataset, which was our intention.
On the other hand, metrics like internal diversity, novelty, uniqueness and validity stay fairly similar, or even become better for the biased models. We can conclude that these metrics are not compromised due to biasing.

The following tables have the metrics for Wasserstein-1 distance of the molecular properties. The metrics are generally on the higher side, however, section 5.1.15 may explain these values

5.1.8 Unbiased models (Wasserstein-1 distance)

Model	QED	SA	logP	weight
Stack RNN	0.089	0.077	0.713	80.801
NTM	0.081	0.108	0.683	81.317
DNC	0.092	0.1	0.837	85.32
GRU	0.076	0.107	0.629	84.541
LSTM	0.081	0.089	0.659	80.278

Vanilla	0.08	0.244	0.836	117.837

Table 41 - Wasserstein-1 distance of properties for unbiased models

The QED distance is fairly similar for all the unbiased models.
The SA distance is similar across all the models except the vanilla RNN which has a higher distance.
The logP distance is fairly varying, with the GRU, LSTM and NTM being on the lower side. While the vanilla RNN and DNC are slightly higher.
The weight distance is similar to the SA, with only the vanilla RNN having a higher distance compared to the others.

5.1.9 DNC biased towards benzene (Wasserstein-1 distance)

Model	QED	SA	logP	weight
Maximized	0.13	0.169	2.734	55.246
Minimized	0.027	0.441	2.652	109.071
Unbiased	0.092	0.1	0.837	85.32

Table 42 - Wasserstein-1 distance of properties for DNC biased towards number of benzene rings

5.1.10 DNC biased towards Log P(Wasserstein-1 distance)

Model	QED	SA	logP	weight
Maximized	0.02	0.266	1.185	80.512
Minimized	0.045	0.26	2.498	91.701
Unbiased	0.092	0.1	0.837	85.32

Table 43 - Wasserstein-1 distance of properties for DNC biased towards Log P

5.1.11 NTM biased towards benzene (Wasserstein-1 distance)

Model	QED	SA	logP	weight
Maximized	0.038	0.681	0.852	63.956

Minimized	0.306	0.105	2.451	67.582
Unbiased	0.081	0.108	0.683	81.317

Table 44 - Wasserstein-1 distance of properties for NTM biased towards number of benzene rings

5.1.12 NTM biased towards Log P(Wasserstein-1 distance)

Model	QED	SA	logP	weight
Maximized	0.03	0.178	1.018	90.101
Minimized	0.029	0.153	2.362	127.346
Unbiased	0.081	0.108	0.683	81.317

Table 45 - Wasserstein-1 distance of properties for NTM biased towards Log P

5.1.13 Stack RNN biased towards benzene (Wasserstein-1 distance)

Model	QED	SA	logP	weight
Maximized	0.307	0.611	1.031	168.87
Minimized	0.044	0.656	2.242	63.338
Unbiased	0.089	0.077	0.713	80.801

Table 46 - Wasserstein-1 distance of properties for Stack RNN biased towards number of benzene rings

5.1.14 Stack RNN biased towards Log P (Wasserstein-1 distance)

Model	QED	SA	logP	weight
Maximized	0.037	0.213	0.797	68.516
Minimized	0.034	0.358	2.029	53.257
Unbiased	0.089	0.077	0.713	80.801

Table 47 - Wasserstein-1 distance of properties for Stack RNN biased towards Log P

The QED generally reduces for the biased models.

The SA and log P increase for the biased models A possible explanation for SA increasing is that the biased molecules are inherently harder to synthesize. Log P increasing is expected as the molecules would inevitably be farther away from the dataset, whether minimized or maximized. The weight is fairly random in nature.

As some of the values were surprisingly low, we ran a control. The generated set in this case was taken from the test set. While the test set was a random sample of 100k molecules from the training set.

5.1.15 Control (Wasserstein-1 distance)

Model	'SNN/Test'	Scaf/Test	QED	SA	logP	weight
Test set	0.579	0.544	0.090	0.152	0.66	79.49

Table 48 - Wasserstein-1 distance of properties for the control set

It can be seen that these values do not differ too much compared to the molecules that were generated by our models.

5.2 Fréchet ChemNet Distance

We have used the Fréchet ChemNet Distance (FCD) as a quantitative measure of the similarity between the generated molecules and the dataset used to train the generator.

**Property - Bias**	**Model**	**FCD**
Unbiased	Vanilla	4.998
	GRU	1.226
	LSTM	0.591
	StackRNN	0.546
	NTM	0.600
	DNC	0.706
logP- Min	StackRNN	10.767

	NTM	8.121
	DNC	6.917
logP- Max	StackRNN	8.804
	NTM	18.137
	DNC	17.892
Benzene- Min	StackRNN	15.189
	NTM	25.989
	DNC	11.736
Benzene- Max	StackRNN	15.133
	NTM	17.227
	DNC	22.534

Table 49 - FCD for unbiased baseline models and biased memory augmented models

As seen from the results above, the unbiased models of the StackRNN, NTM and DNC have a very low FCD to the training dataset used to train the generators. This indicates that these models, when only optimized towards validity of the SMILES format, are capable of learning and producing diverse molecules which possess chemical and biological properties
similar to already known molecules (the training dataset).

When examining the values of FCD for all the biased models, it is much higher than the unbiased models, as expected. The models are biased towards producing molecules with a certain property, and hence are expected to have a larger dissimilarity with the overall training dataset.

5.3 Common string percentage with additional data

5.3.1 Common string percentage

Following is the overlap percentage against 86.8 million molecules.

**Model**	**Common string percentage**
Dnc Unbiased	9.9209
NTM Unbiased	11.2469

Stack Unbiased	8.4767
Baseline GRU unbiased	4.9516
Baseline vanilla unbiased	6.4406
Baseline LSTM unbiased	6.9882
DNC Min logP	7.9348
NTM Min logP	9.0352
Stack Min logP	3.6614
DNC Max logP	1.9231
NTM Max logP	2.8433
Stack Max logP	3.5098
DNC Min Ben	5.0847
NTM Min Ben	2.4175
Stack Min Ben	3.9902
DNC Max Ben	3.1020
NTM Max Ben	9.5913
Stack Max Ben	1.0338

Table 50 - Common string percentage

5.3.2 Data Sources

ChemBL	From existing dataset
CheBI	https://ftp.ebi.ac.uk/pub/databases/chebi/
MOSES dataset	https://media.githubusercontent.com/media/molecularsets/moses/master/data/dataset_v1.csv
Emolecules	https://downloads.emolecules.com/free/2021-10-01/
Mcule	https://mcule.com/database/
Comptox	ftp://newftp.epa.gov/COMPTOX/Sustainable_Chemistry_Data/Chemistry_Dashboard/DSSTOX_MS_Ready_Chemical_Structures.zip
DrugBank	https://go.drugbank.com/releases/latest#open-data

Drug Central	https://drugcentral.org/download

Table 51 - Data Sources

5.4 Mean and Std Dev of property values

The differences in performance between the three RNNs are statistically significant. Below are the results acquired by generating five samples of strings each consisting of six thousand strings for every RNN model. The results contain the standard deviation of the means and average of mean values of a property for all the samples.

5.4.1 LogP

Model	Mean of means	Std of means
StackRNN unbiased	2.8340	0.0107
StackRNN maximized	4.3418	0.0387
StackRNN minimized	1.5179	0.0356
NTM unbiased	2.8629	0.0176
NTM maximized	4.5650	0.0190
NTM minimized	1.1833	0.0085
DNC unbiased	2.7083	0.0203
DNC maximized	4.7303	0.0125
DNC minimized	1.0473	0.0159

Table 53 - Mean and Standard deviation of Log P values of generated strings

5.4.2 Benzene Rings

Model	Mean of means	Std of means
StackRNN unbiased	1.1610	0.0024
StackRNN maximized	2.0008	0.0155
StackRNN minimized	0.4044	0.0039
NTM unbiased	1.1568	0.0096
NTM maximized	2.2405	0.0067
NTM minimized	0.1566	0.0050
DNC unbiased	1.1058	0.0093
DNC maximized	2.6223	0.0130
DNC minimized	0.1603	0.0052

Table 54 - Mean and Standard deviation of number of benzene rings of generated strings

From the results for the NTM and DNC, we can see that they perform statistically significantly (> 2 standard deviation) better than the stack RNN.

5.5 Mean and Std Dev of valid percentage

5.5.1 Unbiased

Model	Mean of valid %	Std of valid %
StackRNN unbiased	92.9806	0.1656
NTM unbiased	95.0428	0.2179
DNC unbiased	94.5667	0.2305
Baseline GRU unbiased	82.4934	0.4497
Baseline vanilla unbiased	32.2255	0.5070
Baseline LSTM unbiased	93.3962	0.1792

Table 55 - Mean and Standard deviation of valid percentage of generated strings for unbiased models

5.5.2 LogP optimized models

Model	Mean of valid %	Std of valid %
StackRNN maximized	89.3981	0.5051
StackRNN minimized	96.8882	0.1084
NTM maximized	95.1118	0.2029
NTM minimized	95.9145	0.3108
DNC maximized	94.3717	0.2873
DNC minimized	92.2473	0.2096

Table 56 - Mean and Standard deviation of valid percentage of generated strings for LogP optimized models

5.5.3 Benzene Rings optimized models

Model	Mean of valid %	Std of valid %
StackRNN maximized	80.8069	0.3251
StackRNN minimized	93.6177	0.1268
NTM maximized	96.1349	0.1135
NTM minimized	89.7500	0.3225
DNC maximized	96.7154	0.2135
DNC minimized	92.6729	0.2176

Table 57 - Mean and Standard deviation of valid percentage of generated strings for benzene ring optimized models

6. Comparison of models with lower computational units

As the above results showed that the LSTM and stack RNN are comparable, we investigate how much the augmented memory helps. We hypothesize that since we used such a large number of LSTM units(1024), the benefit of extra memory is not seen. Thus, we experiment with lower number of LSTM units/memory. We compare only the LSTM with the stack RNN as the stack RNN uses LSTM units, and it performed the worst out of the augmented models. Hence, the stack RNN can be considered the smallest improvement over the baseline LSTM (as opposed to the NTM and DNC which have some extra capabilities).
The number of LSTM units we present are 16, 32, 64 and 128.
The hyperparameters used are the same as mentioned in Section 2, apart from num_units(nu) and stack_width(sw).
The data shown in the tables is for validity percentage, and a constant number of LSTM units. I.e. the num_units for the stack and the number of LSTM units are the same.
We generate around 1300 strings at every 5000th iteration till 45000. (This was the same as the models with higher memory).

6.1 Validity percentage for models with 16 units

Iteration number	LSTM	Stack RNN with sw = 4	Stack RNN with sw = 8	Stack RNN with sw = 16
5000	4.381245	5	3.917051	4.37788
10000	9.769231	9.384615	11.30769	10.53846
15000	13.75865	13.23077	14.23077	19.23077
20000	13.84615	16.69231	20.61538	18.15385
25000	20.76923	18.76923	21.53846	17.69231
30000	17.76923	19.15385	24.84615	22.76923
35000	23.15385	24.15385	26.84615	28.15385
40000	20.53846	23.92308	32.76923	27.69231
45000	23.84615	23.07692	25.23077	31.69231

Table 58 - Validity percentage for LSTM and Stack RNN models with 16 computational units

6.2 Validity percentage for models with 32 units

Iteration number	LSTM	Stack RNN with sw = 32	Stack RNN with sw = 512
5000	18.07692308	22.44427364	13.38461538
10000	27.53846154	29.84615385	33.92307692
15000	34.92307692	36.35664873	36.07692308
20000	41.69230769	45.76923077	40.84615385
25000	38.46153846	46.76923077	47.92307692
30000	39.46153846	53.07692308	56.46153846
35000	44	51.61538462	50.15384615
40000	47.69230769	49.69230769	56.07692308
45000	46.69230769	54.84615385	58.53846154

Table 59 - Validity percentage for LSTM and Stack RNN models with 32 computational units

6.3 Validity percentage for models with 64 units

Iteration number	LSTM	Stack RNN with sw = 64
5000	38.76923077	44.04304381
10000	51.38461538	52.61538462
15000	58.69230769	64.53846154
20000	65.07692308	69.84615385
25000	66	70.61538462
30000	66	65.69230769
35000	67.76923077	73
40000	69.92307692	68.23076923
45000	70.84615385	75.15384615

Table 60 - Validity percentage for LSTM and Stack RNN models with 64 computational units

6.4 Validity percentage for models with 128 units

Iteration number	LSTM	Stack RNN with sw = 32	Stack RNN with sw = 128	Stack RNN with sw = 512
5000	59	56.38461538	62.84615385	68.384615
10000	72.15384615	72.61538462	70.07692308	68.076923
15000	73	75.38461538	78.23076923	75.307692
20000	76.92307692	72.76923077	79.38461538	77.692308
25000	78.46153846	78.53846154	77.61538462	79.538462
30000	77.46153846	77.07692308	79.84615385	83.076923
35000	79.15384615	80.46153846	80.92307692	81.692308
40000	81.69230769	82.76923077	83	84
45000	85.84615385	85.30769231	81.69230769	79.923077

Table 61 - Validity percentage for LSTM and Stack RNN models with 128 computational units

From the above tables and our earlier results, we can see the following. From 16 units to 64 units, augmented memory does help in increasing validity percentage. However, for 128 units, the increase in stack memory seems to have no effect, with all the models performing similarly. Along with our results for the higher memory models where the LSTM performed comparably to the stack RNN, we can conclude for our dataset, that beyond a certain number of LSTM units(128 in our case), the benefit of augmented memory is negligible.

7. 2-D representation of molecule generated

Below are the 2-D structures of a sample of the molecules generated that have been optimized towards minimizing the value of logP. The synthetic accessibility scores of the molecules are also calculated.

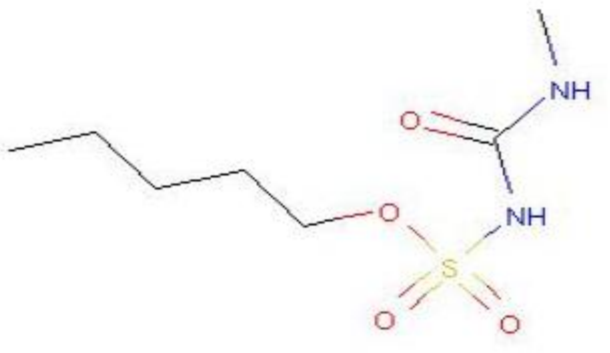

LogP Value: 0.36699999999999955
SA_score: 2.56
SMILES String: CNC(=O)NS(=O)(=O)OCCCCC

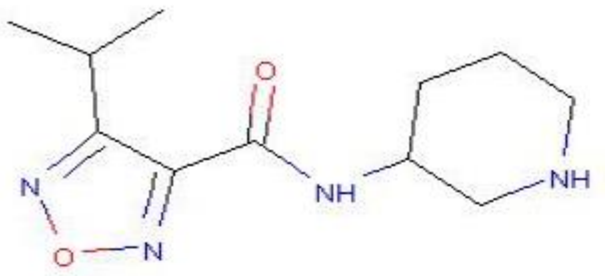

LogP Value: 0.6747999999999998
SA_score: 3.23
SMILES String: CC(C)c1nonc1C(=O)NC1CNCCC1

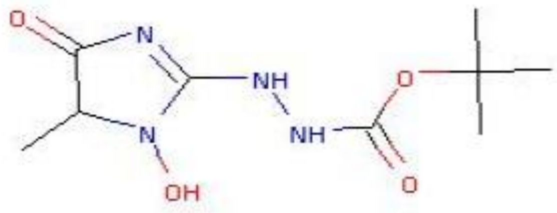

LogP Value: -0.00840000000000063
SA_score: 3.71
SMILES String: CC1N(O)C(NNC(=O)OC(C)(C)C)=NC1=O

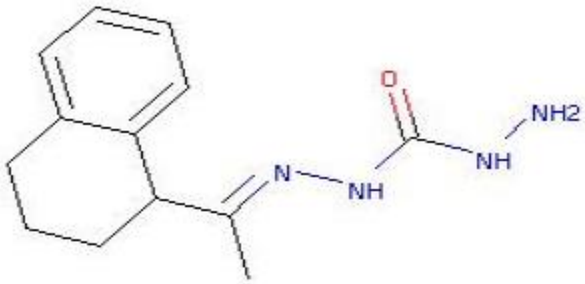

LogP Value: 1.6553
SA_score: 3.11
SMILES String: NNC(=O)NN=C(C)C1c2ccccc2CCC1
