# Supplementary material for: Memory augmented recurrent neural networks for de-novo drug design
Source: PLoS One. 2022 Jun 23;17(6):e0269461. doi: 10.1371/journal.pone.0269461 (PMC9223405; doi:10.1371/journal.pone.0269461)
Supplement: S4 File — (ZIP) [file pone.0269461.s004.zip › PONE-D-21-26539R2_ReproducibleResearchRepository3_Output/2D Structural Representation.zip!/2D Structural Representation/templates/home_page.html]

De novo drug design


PW20GS103

# De Novo Drug Generation

---

Click the below icon to generate entirely new molecules!

De Novo Drugs
  
Molecules not present in
  
any known database


Entire databases generated
  
Large amount of molecules
  
for further analysis


Optimized Towards Target
  
Generate drugs with
  
desired properties
