# Supplementary material for: Memory augmented recurrent neural networks for de-novo drug design
Source: PLoS One. 2022 Jun 23;17(6):e0269461. doi: 10.1371/journal.pone.0269461 (PMC9223405; doi:10.1371/journal.pone.0269461)
Supplement: S4 File — (ZIP) [file pone.0269461.s004.zip › PONE-D-21-26539R2_ReproducibleResearchRepository3_Output/2D Structural Representation.zip!/2D Structural Representation/templates/mol_page.html]

De Novo Drug Generation


De novo Drug Design


- Generator
- Bias
- Molecular Property

Number of Molecules

Generate Stings

##### Selected Generator

##### Selected Bias

##### Selected Property

##### Invalid molecules
